# *Pseudomonas aeruginosa* two-component system CprRS regulates HigBA expression and bacterial cytotoxicity in response to LL-37 stress

**DOI:** 10.1371/journal.ppat.1011946

**Published:** 2024-01-10

**Authors:** Yingjie Song, Siping Zhang, Ninglin Zhao, Cheng Nong, Yongxing He, Rui Bao

**Affiliations:** 1 College of Life Science, Sichuan Normal University, Chengdu, China; 2 Ministry of Education Key Laboratory of Cell Activities and Stress Adaptations, School of Life Sciences, Lanzhou University, Lanzhou, China; 3 Center of Infectious Diseases, Division of Infectious Diseases in State Key Laboratory of Biotherapy, West China Hospital, Sichuan University, Chengdu, China; University of North Carolina at Chapel Hil, UNITED STATES

## Abstract

*Pseudomonas aeruginosa* is a highly pathogenic bacterium known for its ability to sense and coordinate the production of virulence factors in response to host immune responses. However, the regulatory mechanisms underlying this process have remained largely elusive. In this study, we investigate the two-component system CprRS in *P*. *aeruginosa* and unveil the crucial role of the sensor protein CprS in sensing the human host defense peptide LL-37, thereby modulating bacterial virulence. We demonstrate that CprS acts as a phosphatase in the presence of LL-37, leading to the phosphorylation and activation of the response regulator CprR. The results prove that CprR directly recognizes a specific sequence within the promoter region of the HigBA toxin-antitoxin system, resulting in enhanced expression of the toxin HigB. Importantly, LL-37-induced HigB expression promotes the production of type III secretion system effectors, leading to reduced expression of proinflammatory cytokines and increased cytotoxicity towards macrophages. Moreover, mutations in *cprS* or *cprR* significantly impair bacterial survival in both macrophage and insect infection models. This study uncovers the regulatory mechanism of the CprRS system, enabling *P*. *aeruginosa* to detect and respond to human innate immune responses while maintaining a balanced virulence gene expression profile. Additionally, this study provides new evidence and insights into the complex regulatory system of T3SS in *P*. *aeruginosa* within the host environment, contributing to a better understanding of host-microbe communication and the development of novel strategies to combat bacterial infections.

## Author summary

*Pseudomonas aeruginosa* is a ubiquitous Gram-negative bacterium that commonly causes fatal infections in immunosuppressed individuals. However, the underlying mechanisms that enable *P*. *aeruginosa* to adapt to capricious environments of the human body are still poorly understood. Recent studies unveil that two-component systems (TCSs) play a vital role in pathogenesis by sensing environmental cues and modulating gene expression in *P*. *aeruginosa*. In this study, we identified the CprRS, one of TCSs in *P*. *aeruginosa*, as an essential virulence factor. The sensor protein CprS directly interacts with human host defense peptide LL-37 and activates the response regulator CprR, which then coordinates the activities of type III secretion system for higher cytotoxicity. Loss of *cprS* or *cprR* significantly impairs bacterial survival in macrophage and host colonization. Collectively, this work contributes to explain the potent role of CprRS in the molecular-level of host-microbe communication to efficiently survival within the human host.

## Introduction

The host innate immune system plays a crucial role in recognizing and eliminating invading bacteria through a variety of mechanisms, including pattern recognition receptors (PRRs) that recognize conserved microbial structures such as lipopolysaccharides and peptidoglycans [1–3]. Upon recognition, the host immune system activates various defense mechanisms to eliminate the invading bacteria. One important component of the host defense is the secretion of antimicrobial peptides (AMPs) [4,5]. Immune system secreted AMPs are small cationic peptides that exhibit broad-spectrum antimicrobial activity against bacteria, fungi, and viruses [6]. They can disrupt the bacterial membrane, inhibit protein synthesis, and modulate immune responses [7]. However, bacteria have evolved various strategies to evade or resist the host innate immune system, such as altering the composition of their outer membrane or producing specific surface molecules that interfere with the recognition by PRRs [1,5,8]. Some bacteria can even secrete proteases that degrade AMPs, or modify their own gene expression to resist the antimicrobial effects of AMPs. Therefore, understanding the mechanisms by which bacteria evade antimicrobial peptides is of great significance.

*Pseudomonas aeruginosa* is a notorious opportunistic pathogen known for its ability to evade host immune defenses, particularly in resisting antimicrobial peptides (AMPs) [9–11]. This bacterium possesses large genome and genetic flexibility that enable it to rapidly adapts to the milieu of the airway, and formation of biofilms promotes persistence and evasion of phagocytic clearance [12,13]. One notable feature is the abundance and diversity of two-component systems (TCS) in *P*. *aeruginosa*, which play a crucial role in regulating its response to host and environmental stimuli, including AMPs [14,15]. Several *P*. *aeruginosa* TCS systems, such as PhoPQ, PmrAB, ColRS, ParRS and CprRS, have been identified to be involved in Polymyxin resistance [15]. They collectively function to sense ions and cationic antimicrobial peptides, regulating the expression of the *arnBCADTEF* operon, which encodes enzymes responsible for modifying lipopolysaccharide (LPS) [16]. This LPS modification enhances resistance not only to host-derived cationic antimicrobial peptides but also to Polymyxin antibiotics. Activation of the sensor protein by various cationic antimicrobial peptides leads to the upregulation of the *arn* operon, facilitating LPS modification. Moreover, recent reports have indicated that these TCS systems may also sense additional diverse cationic peptides, regulating bacterial resistance and virulence [17,18]. For example, studies have demonstrated that the CprRS as well as ParRS system can be stimulated by human opioid peptide dynorphin, resulting in increased virulence factor production and adaptive resistance in *P*. *aeruginosa* [17]. Interestingly, elevated levels of dynorphin, under host stress, have been found to trigger virulence in *P*. *aeruginosa* through an unknown pathway [17]. These findings suggest that the CprRS system may play a role in host-microbe communication via small molecule signals. However, the molecular mechanisms underlying these observations remain unclear.

Building upon these compelling discoveries, our study provides further evidence demonstrating the direct recognition of human host defense peptide LL-37 by the sensor protein CprS, which subsequently modulates the expression of multiple virulence genes. Specifically, we have elucidated that phosphorylation of the response regulator CprR at residue D53 by CprS significantly enhances the expression of *higB* in the presence of LL-37. Through comprehensive quantitative proteomics and transcriptional analysis, we have established the indispensable role of the activated toxin HigB in mediating the expression of T3SS genes, thereby ensuring complete virulence and bacterial cytotoxicity. Moreover, we have demonstrated that the deletion of *cprS* or *cprR* abolishes *P*. *aeruginosa*’s ability to suppress the expression of proinflammatory cytokines in macrophages, as well as its virulence in the *Galleria mellonella* model. Collectively, our study highlights the critical function of the CprRS system in interkingdom communication, serving as an essential strategy for sensing human immune responses and coordinating *P*. *aeruginosa* virulence.

## Results

### Functional and proteomic characterization of CprRS in *P*. *aeruginosa*

**Fig 1:**
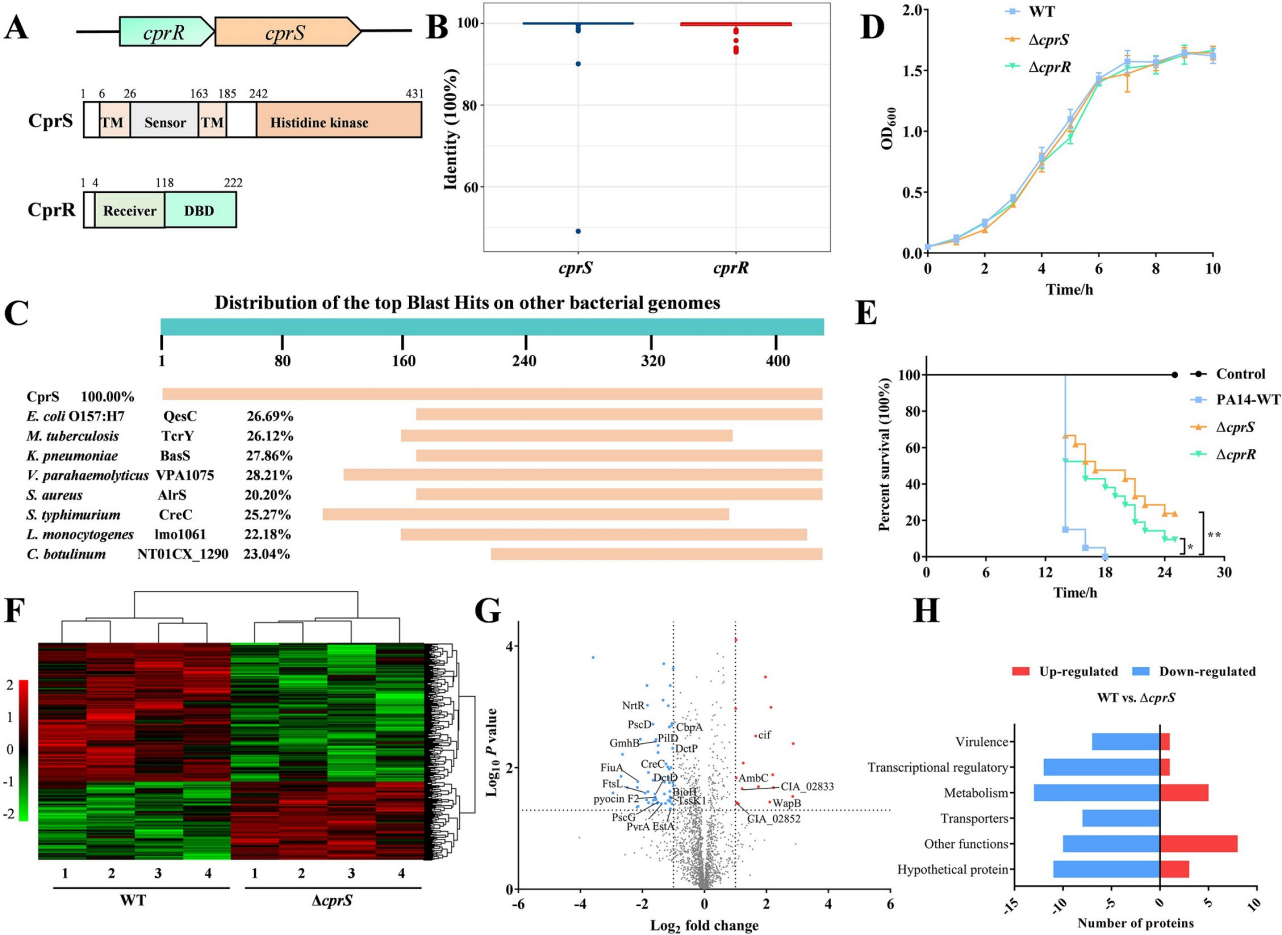
CprRS is conserved in *P*. *aeruginosa* and contributes to its full virulence. (A) Schematic representation of CprRS system genes and proteins. (B) Conservation analysis of *cprRS* genes in 872 *P*. *aeruginosa* strains. Dots represent outliers from the respective groups. (C) The top sequence Blast hits of CprS on other pathogen genomes by NCBI. The identities to CprS are also marked. (D) Growth curves of WT and mutants on LB medium. (E) Measurement virulence of the WT and mutant strains in a *Galleria mellonella* infection model. Each *G*. *mellonella* was injected with 10 μL of *P*. *aeruginosa* dilution (5 × 10^3^ CFU/mL), and the PBS-injected larvae were the negative control. The larvae were monitored for 24 h after the infection (Mantel-Cox test for statistics, *P < 0.05). (F) Hierarchical clustering of the z-scored extracted ion chromatogram was used to evaluate the reproducibility of the proteome quantification in WT and Δ*cprS* strains. (G) Volcano plot displaying the proteomic profiles of WT and Δ*cprS* strains. The significantly up- and down-regulated proteins are labeled with red and cyan, respectively. The significant expressed proteins are also categorized by functional category in (H). Student’s *t*-test was used to assess the significance of differential expression of proteins (DEPs) between two groups. Proteins that have significance level of *P* < 0.05 and fold change >1.5 or < –1.5 were considered as DEPs.

According to the Prokaryotic 2-Component Systems (P2CS) databases, over 60 TCSs have been identified in the complete genome of *P*. *aeruginosa* PAO1 [19]. The sensor protein CprS, consisting of 431 amino acids, contains two transmembrane domains, a distinct extracellular sensor domain for signal recognition, and an intracellular histidine kinase domain (Fig 1A). The corresponding response regulator, CprR, consists of an N-terminal regulatory receiver domain and a C-terminal OmpR/PhoB-type domain for DNA binding (Fig 1A). To assess the conservation of the CprRS system, we analyzed 872 clinical isolates of *P*. *aeruginosa* obtained from the Pseudomonas Genome DB using the DIAMOND BLASTX method [20]. The results revealed a high conservation of both *cprS* and *cprR* among different *P*. *aeruginosa* strains (Fig 1B). Intriguingly, orthologs of CprS were rarely found in other pathogenic bacteria, and the sensor domain displayed no similarity to other histidine kinases of TCSs, highlighting its potential physiological importance in *P*. *aeruginosa* (Fig 1C). Similar to many RR, CprR can be phosphorylated by CprS in the presence of ATP, while the CprR D53A mutant, which contains a conserved site for putative modification, failed to exhibit this behavior, indicating that CprS acts on the D53 site of CprR (S1A and S1B Fig).

To further investigate the potential involvement of CprRS in vital metabolic pathways and virulence, we generated Δ*cprS* and Δ*cprR* mutants and evaluated their effects on growth rate. While no noticeable differences were observed between the wild-type (WT) strain and the mutants in LB medium (Fig 1D), survival studies demonstrated that the loss of *cprS* or *cprR* significantly reduced *G*. *mellonella* mortality compared to the WT strain. By 18 h, Δ*cprS* and Δ*cprR* led to death rates of 50.0% and 60.0%, respectively, while the WT group exhibited near 100% mortality (Fig 1E). Due to the limited research conducted on the signaling pathways associated with CprRS, we performed global proteomic profiling of the Δ*cprS* and WT strains using label-free quantitative proteomics to gain insights into how CprRS may impact bacterial virulence (Fig 1F). Comparative analysis revealed a decreasing trend in the overall protein levels of *P*. *aeruginosa* Δ*cprS*, with 61 down-regulated proteins and 18 up-regulated proteins (Fig 1G and Table 1). Among the down-regulated proteins, ∼22% decreases were observed for basal metabolism including amino acid synthesis (MurI, SpeA, GltD, and ItaA), ribonucleotide metabolism (PurK, RibC, and NrdJa), and carbon metabolism (GmhB, FndG), which suggested that loss of *cprS* may affect some essential metabolisms. The proteins involved in transcriptional regulation and transportation also exhibited significant decreases, such as TonB-dependent receptor FiuA, C4-dicarboxylate-binding periplasmic protein DctP, C4-dicarboxylate transport transcriptional regulatory protein DctD, multidrug ABC transporter CIA_03021 (Fig 1H). Additionally, proteins associated with virulence were diminished, including Prepilin leader peptidase PilD and assembly protein PA3688, virulence regulator PvrA [21], pyocin F2, T3SS export proteins PscD and PscG, and the Type VI secretion system (T6SS) baseplate component TssK1 [22] (Fig 1H), consistent with the results from infection models. Among the proteins exhibiting increased expression levels, most encoded hypothetical proteins with unclear functions (Fig 1H). Collectively, these findings provide strong evidence for the high conservation of the CprRS system in *P*. *aeruginosa* and its critical role in virulence.

**Table 1:** Significant downregulated proteins in *cprS*-mutant compared with WT.

Locus in PAO1	Locus in PA14	Protein name and Functions	Fold changes (log_2_) *cprS*-KO /WT	Student’s T-test *p*-value
PA4662	CIA_00037	Glutamate racemase MurI	-1.85595	0.000444
PA4577	CIA_00129	DksA C4-type domain-containing protein	-1.10466	0.000444
PA4571	CIA_00135	cytochrome c	-1.0095	0.019971
	CIA_00184	hypothetical protein	-1.65071	0.034178
PA4528	CIA_00287	Prepilin leader peptidase/N-methyltransferase PilD	-1.5617	0.003437
PA4419	CIA_00398	cell division protein FtsL	-1.91464	0.026204
PA4371	CIA_00447	Cytochrome c domain-containing protein	-1.65775	0.016008
PA0863	CIA_00757	Probable oxidoreductase	-1.56208	0.033593
PA0937	CIA_00836	hypothetical protein	-1.00677	0.000234
PA1015	CIA_00916	IclR family transcriptional regulator	-1.51766	0.038518
PA1137	CIA_01043	Probable oxidoreductase	-2.06519	0.003396
PA1513	CIA_01430	Recombination protein RecR	-2.69237	0.013884
PA1648	CIA_01568	Probable oxidoreductase	-1.82312	0.024699
PA1713	CIA_01643	type III export protein PscD	-1.25514	0.038672
PA1720	CIA_01646	type III export protein PscG	-1.66436	0.001928
PA1771	CIA_01698	EstX	-1.49711	0.037266
PA1790	CIA_01717	tRNA-(ms [2] io [6]A)-hydroxylase	-2.13791	0.043151
PA1996	CIA_01934	Peptidyl-prolyl cis-trans isomerase C1 PpiC1	-1.80193	0.0378
	CIA_02165	hypothetical protein	-1.08081	0.009901
PA2289	CIA_02231	TonB-denpendent receptor FiuA	-2.1495	0.016945
PA2484	CIA_02429	TetR family transcriptional regulator	-3.59182	0.000154
PA2692	CIA_02692	transcriptional regulator	-2.48587	0.02128
PA2957	CIA_02992	Virulence regulator PvrA	-1.38643	0.038915
PA2973	CIA_03008	peptidase S49	-1.04834	0.0392
PA2979	CIA_03014	3-deoxy-manno-octulosonate cytidylyltransferase KdsB	-1.59888	0.033637
PA2986	CIA_03021	multidrug ABC transporter substrate-binding protein	-1.40285	0.014567
PA3055	CIA_03091	Antitoxin HipB of type II toxin-antitoxin system	-2.16666	0.021098
PA3213	CIA_03249	Mce/MlaD domain-containing protein	-1.4936	0.004312
PA3247	CIA_03304	M18 aminopeptidase in mucin utilization ApeB	-1.13949	0.010567
PA3626	CIA_03682	tRNA pseudouridine synthase D TruD	-1.01474	0.0176
PA3688	CIA_03744	Pilin assembly protein	-1.16631	0.034608
	CIA_03936	hypothetical protein	-1.11363	0.035795
PA4055	CIA_04155	Riboflavin synthase alpha chain RibC	-1.49465	0.005636
PA4059	CIA_04159	Methyltransferase	-1.66981	0.03972
PA0634	CIA_04420	pyocin F2	-1.58499	0.031002
PA0599	CIA_04454	DUF3530 family protein	-1.80607	0.011963
PA0523	CIA_04534	Nitric oxide reductase subunit C NorC	-1.16569	0.000956
PA0517	CIA_04540	Cytochrome c55X NirC	-1.04545	0.001868
PA0502	CIA_04555	biotin biosynthesis protein BioH	-1.11665	0.02449
PA0464	CIA_04592	sensor histidine kinase CreC	-1.24129	0.008659
PA0144	CIA_04931	Nucleoside 2-deoxyribosyltransferase	-1.86685	0.033868
PA0079	CIA_04998	Type VI secretion system baseplate component TssK1	-1.10562	0.030308
PA0024	CIA_05056	Oxygen-dependent coproporphyrinogen-III oxidase HemF	-1.14831	0.01499
PA0006	CIA_05074	D-glycero-beta-D-manno-heptose-1,7-bisphosphate 7-phosphatase GmhB	-1.57691	0.00368
PA5565	CIA_05085	tRNA uridine 5-carboxymethylaminomethyl modification enzyme MnmG	-1.08679	0.031859
PA5166	CIA_05139	C4-dicarboxylate transport transcriptional regulatory protein DctD	-1.59323	0.027239
PA5497	CIA_05153	Vitamin B12-dependent ribonucleotide reductase NrdJa	-1.29239	0.027055
PA5425	CIA_05226	N5-carboxyaminoimidazole ribonucleotide synthase PurK	-1.31462	0.000195
PA5413	CIA_05238	Low specificity L-threonine aldolase ltaA	-1.29148	0.017305
PA5221	CIA_05439	Probable FAD-dependent monooxygenase	-1.12814	0.017697
PA5167	CIA_05495	C4-dicarboxylate-binding periplasmic protein DctP	-1.02455	0.004803
PA5112	CIA_05554	esterase EstA	-1.09764	0.047568
PA5035	CIA_05632	Glutamate synthase small chain GltD	-1.05859	0.002013
PA4970	CIA_05698	hypothetical protein	-1.33675	0.000776
PA4916	CIA_05754	NUDIX hydrolase NrtR	-1.8394	0.00094
PA4906	CIA_05764	GntR family transcriptional regulator	-1.18633	0.009715
PA4841	CIA_05831	NUDIX hydrolase	-2.64446	0.006042
PA4839	CIA_05833	Biosynthetic arginine decarboxylase SpeA	-1.41269	0.01533
PA4812	CIA_05862	Formate dehydrogenase-O, major subunit FdnG	-2.17393	0.044502
PA4795	CIA_05882	peroxiredoxin	-2.94962	0.026085
PA4704	CIA_05976	cAMP-binding protein A CbpA	-1.13169	0.002133

### The sensor domain of CprS is required for the LL-37 recognition

**Fig 2:**
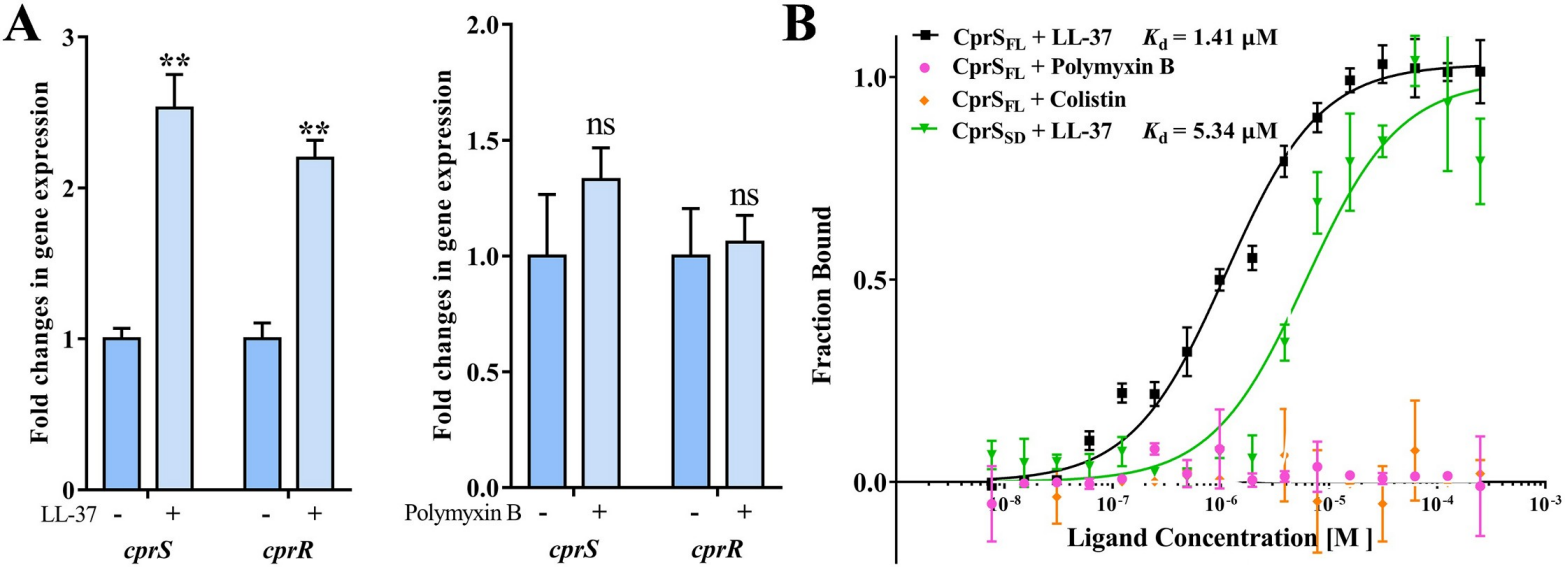
CprS specifically senses and responds to LL-37 signal. (A) The expression levels of *cprS* and *cprR* treated with LL-37 and Polymyxin B were measured by qRT-PCR, respectively. The *oprL* gene was used as a normalizer. (B) The binding affinities of CprS_FL_ and CprS_SD_ toward different cationic peptides were measured with MST. The final protein concentration was 100 nM and cationic peptides have 16 doubling dilutions started from 500 μM. The experiment was repeated three times.

The involvement of the CprRS system in sensing and responding to the stimulation of host AMPs, such as LL-37, has been implicated [23]. However, direct association between the two has yet to be verified. To address this, we conducted quantitative real-time PCR (qPCR) analysis to examine the transcriptional levels of *cprS* and *cprR* under LL-37 conditions. Our findings revealed a significant upregulation of both genes, with *cprS* exhibiting a 2.5-fold increase and *cprR* showing a 2.2-fold increase compared to the control group without LL-37 (Fig 2A). Additionally, we investigated whether Polymyxin B, cationic antimicrobial peptides commonly used for treating *P*. *aeruginosa* infections in clinical settings [24], has any impact on the expression of *cprS* and *cprR*. Interestingly, no significant change in expression was observed under Polymyxin B treatment (Fig 2A). This observation demonstrates that the transcriptional upregulation of the CprRS system is a direct response specifically to LL-37-induced stress.

Subsequently, we expressed and purified the CprS protein, along with its periplasmic sensor domain CprS_SD_ (residues 28–163), and conducted an in vitro direct interaction analysis between CprS and diverse cationic peptides using Microscale Thermophoresis (MST). The results obtained from MST revealed that the human host defense peptide LL-37 exhibited an affinity of 1.41 μM towards the full-length CprS (CprS_FL_), while its affinity towards the CprS sensor domain (CprS_SD_) was slightly lower, approximately around 5.34 μM (Fig 2B). These findings suggest that CprS is capable of directly recognizing LL-37, with its sensor domain playing a significant role in the recognition process. We also investigated the interaction between CprS and Polymyxin B and colistin. Notably, no binding was observed between CprS and Polymyxin B or colistin (Fig 2B), which highlights the specificity of CprS towards LL-37.

### Critical roles of CprRS in the regulation of resistance and virulence-related genes in response to LL-37 stimuli

**Fig 3:**
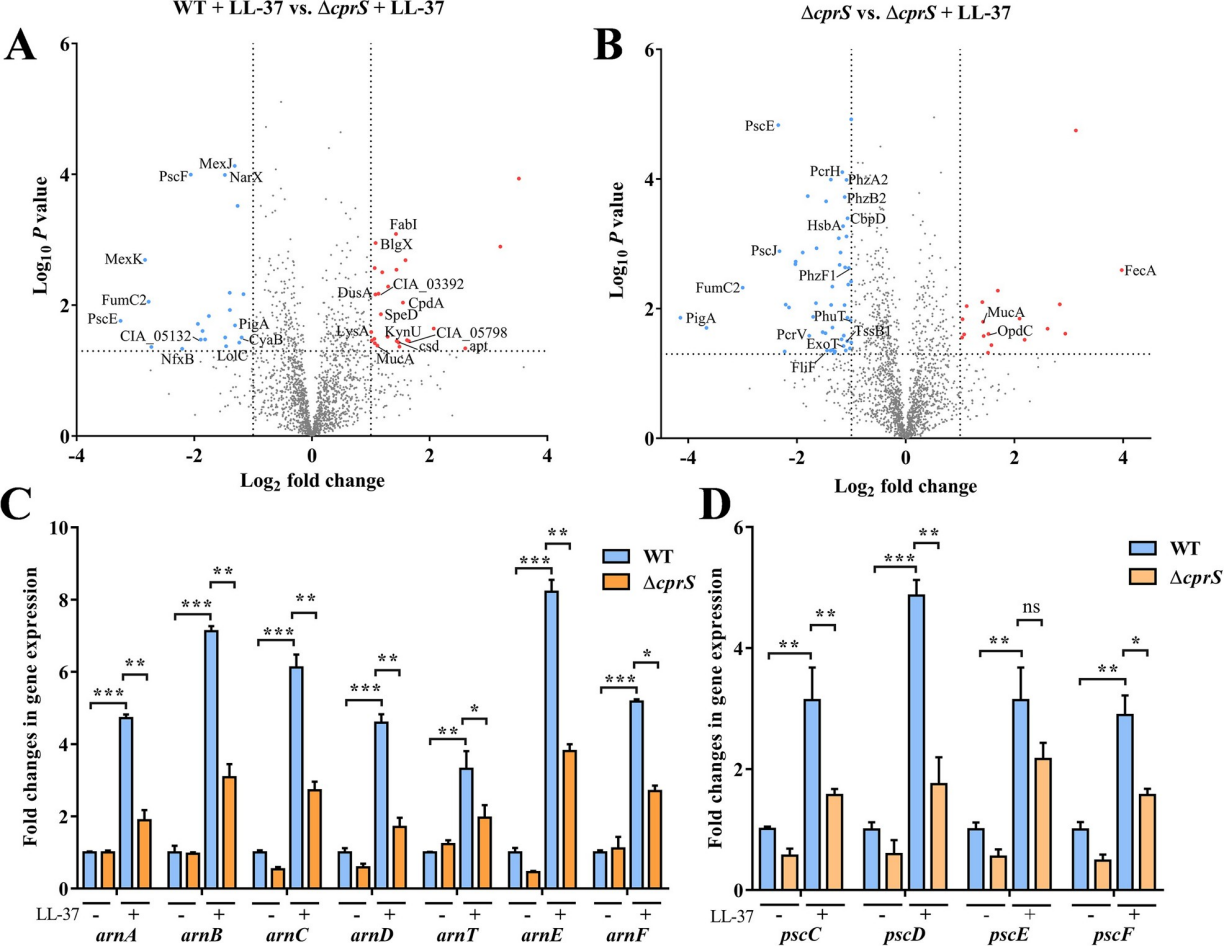
CprRS system senses LL-37 and regulates the expression of genes associated with virulence factors and drug resistance. (A) Volcano plot displaying the proteomic profiles of WT and Δ*cprS* strains after LL-37 treatment. The significantly up- and down-regulated proteins are labeled with red and cyan, respectively. (B) Volcano plot displaying the proteomic profiles of Δ*cprS* strains before and after LL-37 treatment. (C)The expression levels of *arn* operon and (D) *pscCDEF* in WT and Δ*cprS* strains were measured with or without LL-37 treatment, respectively. Error bars indicate the means ± SD of three independent experiments. *P <0.05; **P < 0.01; ***P < 0.001. ns, no significance.

In order to gain further insights into the role of CprS in sensing LL-37 in *P*. *aeruginosa*, we conducted a comparative proteomic analysis between the Δ*cprS* mutant and wild-type (WT) strains under LL-37 treatment. (Figs 3A and S2A and Table 2). Comparing the down-regulated proteins in the *cprS* mutant to the WT strain, we observed that a majority of these proteins belonged to transporters or virulence factors. Notably, multidrug-resistant proteins such as MexK, MexJ, CIA_03021, as well as virulence-associated proteins including PscE, PscF, FumC2, and PigA, were found to be down-regulated (Fig 3A). This suggests that the absence of functional CprS impacts the expression of these proteins, potentially affecting the transport of molecules and compromising virulence. Conversely, the up-regulated proteins in the Δ*cprS* mutant were primarily enzymes associated with metabolic processes. Among these were periplasmic beta-glucosidase BlgX, adenine phosphoribosyltransferase apt, Enoyl-[acyl-carrier-protein] reductase [NADH] FabI, and Diaminopimelate decarboxylase LysA (Figs 3A and S2A). The upregulation of these enzymes indicates a potential compensatory response in metabolic pathways, possibly aimed at maintaining cellular homeostasis in the absence of CprS-mediated LL-37 sensing. Furthermore, we also observed an upregulation of MucA in the *cprS* mutant. MucA negatively regulates the production of alginate, which contributes to virulence and drug resistance [25]. This upregulation suggests that the absence of functional CprS may lead to an altered regulation of alginate production, potentially impacting the virulence and drug resistance mechanisms of *P*. *aeruginosa* (Fig 3A). Additionally, we conducted an analysis of the differentially expressed proteins in Δ*cprS* before and after treatment with LL-37. Our findings revealed a significant decrease in the expression of several proteins directly involved in virulence processes upon stimulation with LL-37. These proteins include PscE, PscH, PscJ, ExoT, PcrV, PcrH, PhzA2, PhzB2, PhzF1, FilF, PigA, and HsbA (Figs 3B and S2B), further supporting the notion that CprRS plays a crucial role in regulating virulence genes following recognition of LL-37.

**Table 2:** Significant downregulated proteins in *cprS*-mutant compared with WT after LL-37 treatment.

Locus in PAO1	Locus in PA14	Protein name and Functions	Fold changes (log_2_) *higA*-KO /WT	Student’s T-test *p*-value
PA4600	CIA_00104	HTH-type transcriptional regulator NfxB	-2.20708	0.04631
PA4470	CIA_00346	Fumarate hydratase FumC2	-2.7776	0.008845
PA0672	CIA_00547	Heme oxygenase PigA	-1.30905	0.020286
	CIA_00715	hypothetical protein	-1.86021	0.024797
PA0926	CIA_00825	hypothetical protein	-2.7315	0.043371
PA1555	CIA_01473	Cbb3-type cytochrome c oxidase subunit CcoP2	-1.265	0.000303
PA1608	CIA_01527	Probable chemotaxis transducer	-1.94121	0.019299
PA1718	CIA_01644	type III export protein PscE	-3.25517	0.017353
PA1719	CIA_01645	type III export protein PscF	-9.06207	0.000101
PA2986	CIA_03021	multidrug ABC transporter substrate-binding protein	-1.46022	0.0423
PA3055	CIA_03091	XRE family transcriptional regulator	-1.3971	0.011846
PA3217	CIA_03253	adenylate and Guanylate cyclase catalytic domain protein CyaB	-1.19478	0.03104
PA3618	CIA_03674	CinA C-terminal domain-containing protein	-1.23689	0.037149
PA3649	CIA_03705	zinc metallopeptidase RseP	-1.75308	0.014601
PA3676	CIA_03732	ultidrug efflux RND transporter permease subunit MexK	-2.83772	0.002031
PA3677	CIA_03733	ultidrug efflux RND transporter permease subunit MexJ	-1.31295	7.40E-05
PA3730	CIA_03785	hypothetical protein	-1.39803	0.006468
PA3878	CIA_03972	two-component system sensor NarX	-1.4803	0.000102
PA0144	CIA_04931	nucleoside deoxyribosyltransferase	-1.81691	0.033387
PA5518	CIA_05132	Probable potassium efflux transporter	-1.89154	0.033531
PA5350	CIA_05306	Rubredoxin-2 AlkG2	-1.47695	0.031127
PA4784	CIA_05893	transcriptional regulator	-1.16402	0.006777

Previous studies have established that the CprRS system in *P*. *aeruginosa* is responsible for sensing various cationic peptides of non-human origin and initiating adaptive resistance [26]. To validate the regulatory role of CprRS in response to LL-37 and its impact on *P*. *aeruginosa* resistance pathways, we performed a measurement of the minimum inhibitory concentrations (MICs) of different antibiotics in Δ*cprR* and Δ*cprS* mutants, both with and without LL-37 treatment. The MIC assay results demonstrated that in the absence of LL-37, the mutants had minimal effects on antibiotic resistance, with only a modest 1 to 2-fold reduction observed in Polymyxin B and Colistin MICs compared to the WT strain (Table 3). However, in the presence of LL-37, significant differences were observed. The mutants exhibited substantial 16 to 32-fold decreases in Polymyxin B and Colistin MICs compared to the WT strain after LL-37 pretreatment (Table 3 and S2C Fig). These findings indicate that the presence of LL-37 potentiates the impact of *cprR* and *cprS* on antibiotic resistance. Consistent with the MIC results, the expression levels of the *arnBCADTEF* operon, known to contribute to cationic antimicrobial peptide resistance [27], were markedly induced under LL-37 conditions in the WT strain, whereas this induction was not observed in the Δ*cprR* and Δ*cprS* mutants (Fig 3C). Furthermore, the *pscDEFG* operon, responsible for exporting effectors via the type III secretion system (T3SS) [28], was notably induced in the WT strain under LL-37 treatment, whereas no such induction was observed in the mutants (Fig 3D). Taken together, these findings provide strong evidence that CprS is directly involved in the recognition of LL-37 and plays a crucial role in the regulation of cationic antimicrobial peptide resistance and virulence.

**Table 3:** MICs to different antibiotics of the WT, the *cprR* and *cprS* mutants.

Antibiotics (μg /mL)	MEM	CEP	TE	GM	CIP	PB	CT	PB + LL-37	CT + LL-37
**WT**	10	0.8	3.2	12.5	2	4	4	64	64
**Δ*cprS***	10	0.8	3.2	12.5	2	2	1	1	0.5
**Δ*cprR***	10	0.8	3.2	12.5	2	2	1	1	1

MEM: Meropenem, CEP: Cephalosporin, TE: Tetracycline, GM: Gentamicin, CIP: ciprofloxacin, PB: Polymyxin B, CT: Colistin.

### CprRS directly regulates the expression of type II TA system HigBA

**Fig 4:**
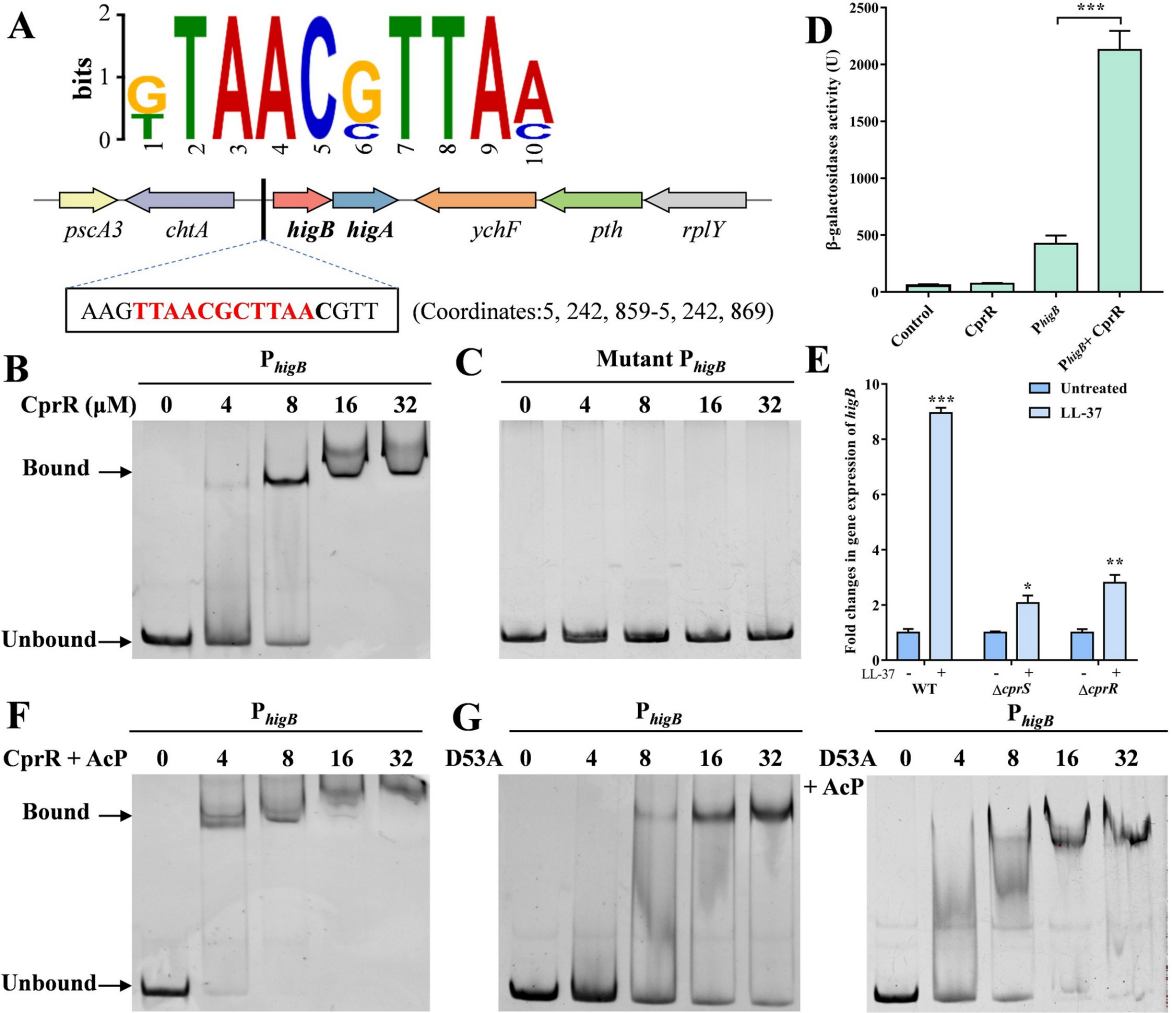
CprR directly activates the expression of type II TA system HigBA. (A) The potential CprR recognition motif and binding site in *higB* promoter. (B) EMSAs showing that CprR binds the promoter region of *higB*. Each reaction mixture contains PCR products of *higB* (1 μM) and the protein concentrations were indicated above the lane. (C) EMSAs of CprR with mutant *higB* promoter. (D) Construction of β-galactosidase reporter system to determine the transcription regulation ability of CprR. The *higB* promoter were cloned ahead of a promoterless *lacZ* gene in pRG970km to construct *lacZ* fusions and then co-transformed into *E*. *coli* BL21 (DE3) with pET22b-*cprR*. The bacteria carrying the vectors were grown in LB medium as OD_600_ reached to 0.6 and supplied with 0.1 mM IPTG for 4 h at 37°C. Then the cells were collected and β-Galactosidase activities were described in methods. (E) AcP treatment enhanced the DNA-binding ability of CprR. (F) The DNA-binding ability of CprR_D53A_ was not affected by AcP.

It is well-established that TCSs in bacteria rely on response regulators (RRs) to modulate the expression of downstream genes upon sensing specific stimuli [15]. In a recent study, a systematic evolution of exponential enrichment assay was utilized to predict the sequence motifs of 371 putative regulators in *P*. *aeruginosa*, including CprR (Fig 4A) [29]. Based on this prediction, a whole-genome search was conducted in *P*. *aeruginosa* strains PAO1 and PA14 to identify potential binding sites of CprR within the non-coding regions located within 300-bp upstream and downstream of protein-coding genes. This search led to the identification of two potential CprR-binding sites in the promoters of *bkdA1* and *higB* genes (S1 Table). The *bkdA1* gene encodes a subunit of an enzyme complex involved in amino acid metabolism [30], while *higB* is part of the type II toxin-antitoxin (TA) system *higBA* [31], which is closely associated with the regulation of the type III secretion system (T3SS) [32], biofilm formation [33], iron uptake [34], and persistence [35,36].

Subsequently, in vitro Electrophoretic Mobility Shift Assay (EMSA) experiments were performed, confirming the binding of purified CprR to the promoter regions of *bkdA1* and *higB* genes, while no binding was observed with mutant promoters lacking the pseudopalindromic sequences (Figs 4B, 4C, S3A, S3B and S3C). Considering the importance of *higB* in bacterial resistance and virulence regulation, further investigations were conducted to examine the regulatory activity of CprRS on *higB* expression. Using a β-Galactosidase reporter system in, it was observed that CprR could activate the *higB* promoter, resulting in a 4.82-fold increase in *E*. *coli* (Fig 4D). When the β-Galactosidase reporter system was employed in *P*. *aeruginosa*, LL-37 was found to exhibit significant positive induction activity on *higB* promoter in WT (S4A Fig). While this positive regulatory activity was markedly reduced in the Δ*cprR* and Δ*cprS* mutants. As expected, the qPCR revealed a significant 8.79-fold increase in *higB* mRNA levels upon LL-37 treatment in the WT strain. However, this induction was substantially attenuated in the Δ*cprR* and Δ*cprS* mutants, with only a 2.12-fold and 2.74-fold increase observed, respectively (Fig 4E). In contrast, under the same conditions, the LL-37 stimulation had limited effects on the expression of *higA*, and no changes were observed due to the mutations in Δ*cprR* or Δ*cprS* (S4B and S4C and S4D Figs). These findings are consistent with previous studies that the *higA* mRNA could be expressed separately from a promoter inside *higB* [31], ensuring rapid activation of the toxin under stressful conditions.

Furthermore, it was discovered that acetyl phosphate (ACP) enhanced the binding of CprR to its target *higB* DNA fragments, whereas the CprS_D53A_ mutant lacking the ability to phosphorylate was unable to exhibit this enhanced binding (Fig 4F and 4G). These collective findings further revealed that *higB* is a critical downstream target directly regulated by CprRS, associated with antibiotic resistance and virulence, while highlighting the role of acetyl phosphate as a modulator of CprS-DNA binding.

### Characterization of the DNA binding domain of CprR by computational modelling and mutagenesis

**Fig 5:**
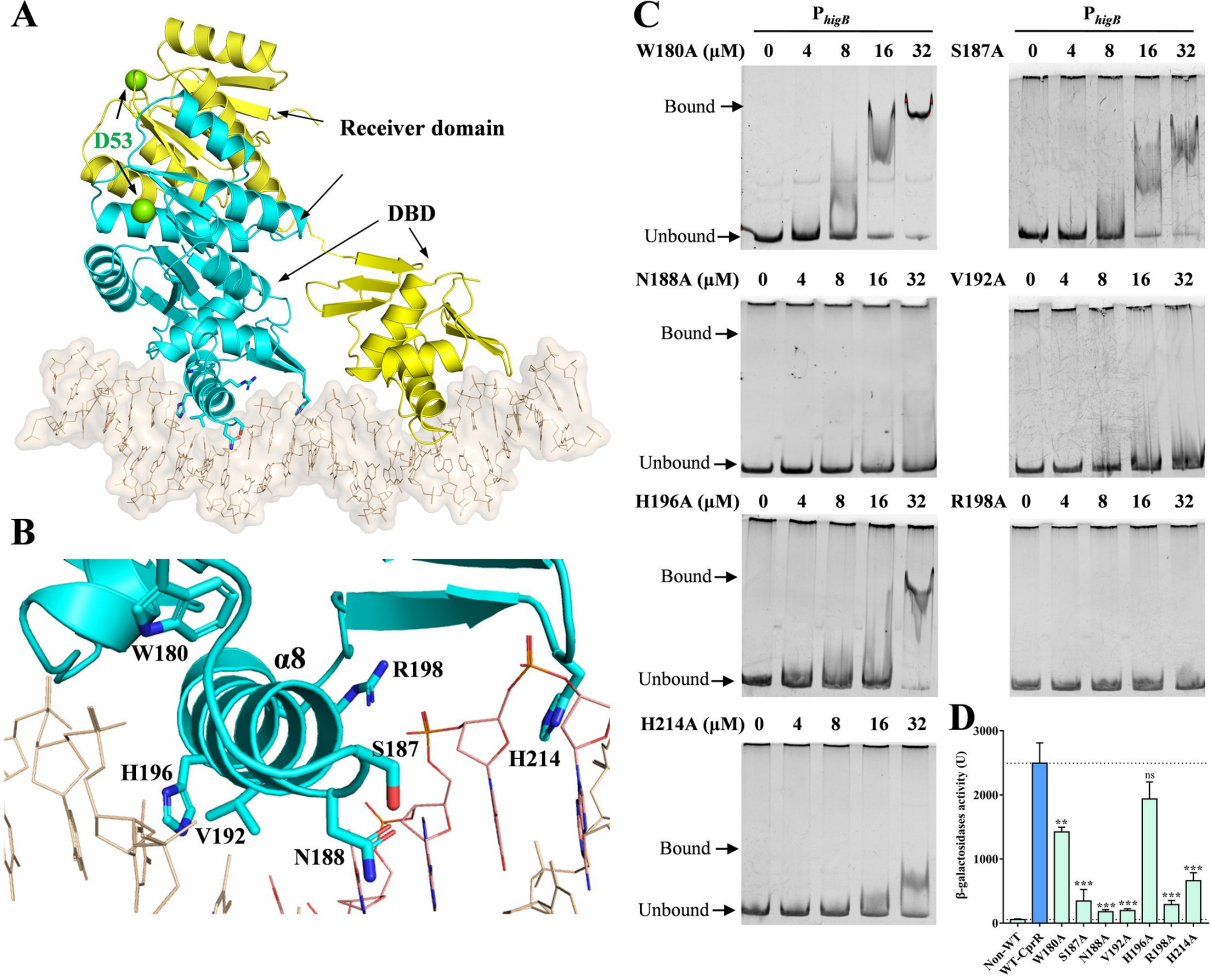
Structural model of CprR-DNA complex. (A) Cartoon presentation of the CprR dimer in complex with the promoter DNA. The two D53 sites are shown as yellow spheres, and the two molecules of CprR dimer are colored in yellow and cyan, respectively. (B) The detailed interaction between DBD and DNA, the residues involved in DNA recognition are shown in sticks. (C) EMSAs of different mutant CprR proteins with *higB* promoter. (D) β-galactosidase reporter system to determine the transcription regulation ability of CprR mutants. *P <0.05; **P < 0.01; ***P < 0.001 by one-way ANOVA statistical test.

Based on the reported structural studies of TCSs, it is known that the transition of RR proteins from the apo state to DNA binding state involves significant conformational changes in the N-terminal receiver domain and the C-terminal DNA binding domain [37,38]. This switching mechanism and structure are conserved among homologous proteins, such as *Klebsiella pneumoniae* PmrA [38] (PDB: 4S04, 36.11% identity with CprR) and *Mycobacterium tuberculosis* PhoP [37] (PDB: 5ED4, 33.03% identity with CprR). To improve our understanding of CprR, we initially generated the structures of its N-terminal and C-terminal domains using AlphaFold [39]. Subsequently, we constructed a model of CprR binding to DNA, based on the structure of *M*. *tuberculosis* PhoP in complex with DNA. The model depicted two CprR molecules bound to a 26-bp double-helix DNA, where the receiver domain of CprR exhibited a (βα)_5_ fold, and its C-terminal DNA binding domain (DBD) featured a winged helix-turn-helix fold (β_5_α_3_) (Figs 5A and S5A).

In the CprR-DNA complex model, the loop between α7 and α8, as well as α8 of the DBD, inserted into the DNA groove. Residues S187, N188, V192, H196, and R198 directly interacted with the DNA phosphate backbone, while H214 extended into the minor groove of the DNA and interacted with the bases. Although W180 did not exhibit direct interaction with the DNA, its presence likely contributed to the stability of the complex based on homologous structures (Fig 5B). Sequence alignment of CprR with its homologues (S5B Fig) revealed high conservation of W180 and R198 among these proteins, while S187, N188, and V192 were moderately conserved. H196 and H214 showed significant differences from other proteins, suggesting their potential role in determining the specificity of the recognition sequence. To validate our molecular model, we performed DNA-binding assays with point mutations (Fig 5C). Consistent with the structural analysis, alanine substitutions of S187, N188, V192, R198, and H214, which directly interact with the DNA phosphate backbone or bases, almost completely abolished the ability of CprR to recognize the target promoter P_*higB*_. On the other hand, the W180A and H196A substitutions retained some DNA binding capacity, but their binding affinities were significantly reduced compared to the wild-type protein (Fig 5C). When these mutations were introduced into the β-Galactosidase reporter assay, we observed varying degrees of reduction in the ability of CprR to upregulate P_*higB*_ activity, and the extent of reduction correlated positively with the weakened DNA binding affinity observed in the EMSA experiments (Fig 5D). These findings provide further support for our molecular model and highlight the importance of these residues in the functional mechanism of CprR in gene regulation.

### CprRS governs the expression of virulence factors by activating the HigBA system

**Fig 6:**
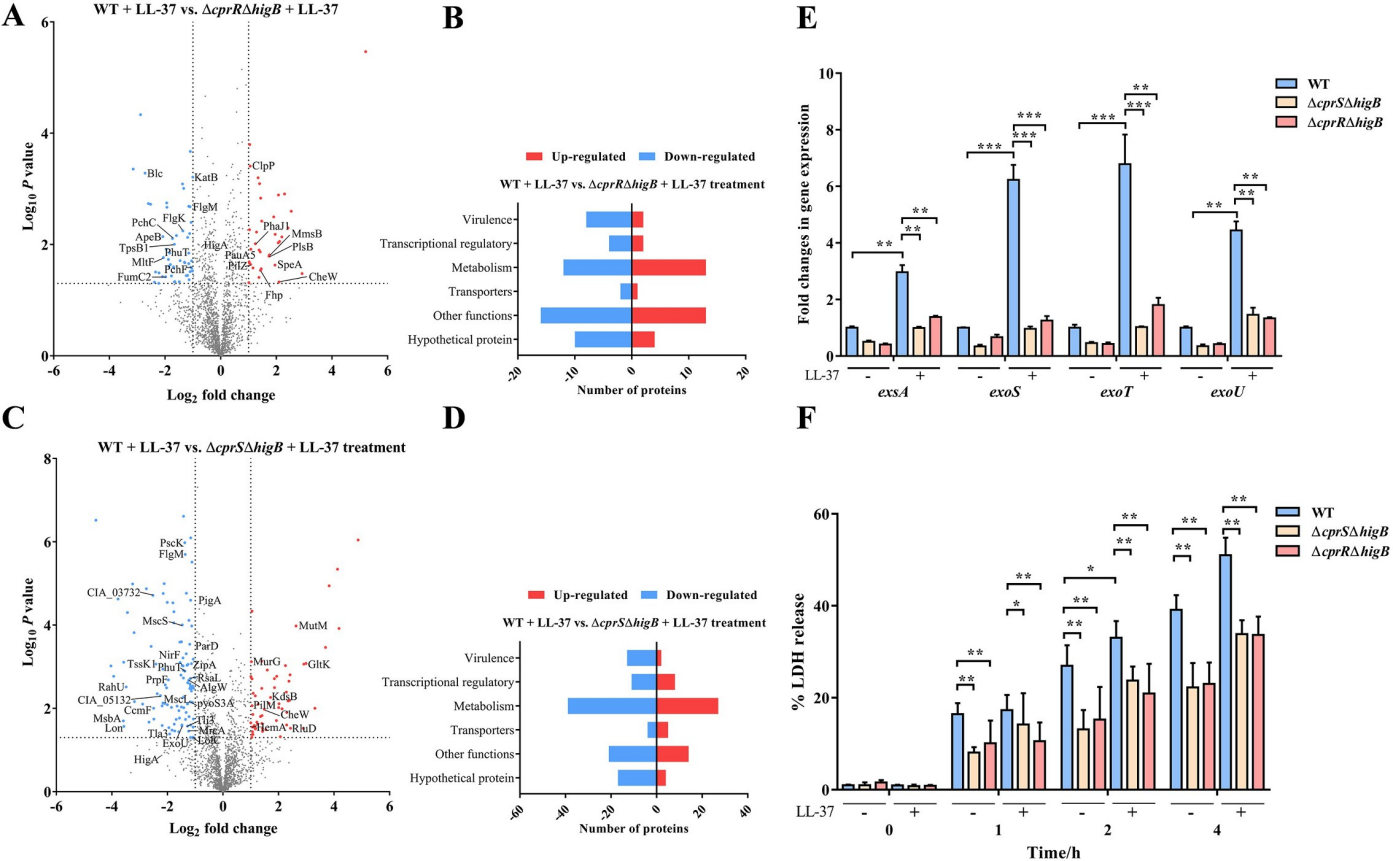
CprRS controls the T3SS expression through HigBA. (A) Volcano plot displaying the proteomic profiles of Δ*cprR*Δ*higB* and (C) Δ*cprS*Δ*higB* compared to WT after LL-37 treatment. (B) and (D) The significant expressed proteins in Fig 5A and 5C are categorized by functional category, respectively. (E) The expression changes of T3SS genes in WT and mutant strains were measured with or without LL-37 treatment, respectively. (F) Raw264.7 cell were infected by different strains at an MOI of 10. At indicated time points, the relative cytotoxicity was determined by the LDH release assay. Error bars indicate the means ± SD of three independent experiments. *P <0.05; **P < 0.01; ***P < 0.001.

To probe the pivotal role of *higB* as a direct downstream target gene regulated by CprRS in mediating the LL-37-induced signaling, we generated Δ*cprR*Δ*higB* and Δ*cprS*Δ*higB* mutants and performed global proteomic profiling in the presence of LL-37 (Figs 6A–6D and S6). A comparative analysis of differentially expressed proteins (DEPs) between Δ*cprR*Δ*higB*/WT and Δ*cprS*Δ*higB*/WT revealed a set of 26 proteins exhibiting similar expression trends, including 20 up-regulated proteins and 6 down-regulated proteins. These DEPs were found to be primarily involved in virulence and iron uptake functions. Specifically focusing on virulence-related proteins, the DEPs in Δ*cprR*Δ*higB*/WT demonstrated varying degrees of down-regulation for pyochelin synthetase PchF, type V protein secretion system complex protein TpsB1, flagellar hook-associated proteins FlgK and FlgL, and outer membrane lipoprotein Blc (Fig 6A and 6B and S2 Table). On the other hand, proteins such as type 4 fimbria biogenesis protein PilZ and Clp protease proteolytic subunit ClpP showed relative upregulation in their expression levels. In Δ*cprS*Δ*higB*/WT, a total of 105 down-regulated proteins and 60 up-regulated proteins were identified (Fig 6C and S3 Table), with approximately 16% of them significantly enriched in virulence functions (Fig 6D). These included subunits of type III secretion system (T3SS) and type VI secretion system (T6SS), such as PscK, PscL, ExoU, Til3, Tla3, CIA_00391, as well as other virulence factors like pyoS3A, RsaL, LolC, NarX, OpgH, PhuT, and PigA [22]. Additionally, Δ*cprS*Δ*higB* exhibited a notable decrease in proteins associated with drug resistance and stress responses, including enzymes and transporters such as serine protease AlgW [40], protease Lon, 2-methylaconitate cis-trans isomerase PrpF, redox-sensitive transcriptional activator SoxR, antitoxin ParD, penicillin-binding protein MrcA, transporter MsbA, and de-N-acetylase DnpA. Taken together with the results presented in Figs 2 and 3, these findings suggest that the diminished virulence observed in Δ*cprR* and Δ*cprS* mutants can be attributed to the pleiotropic effects resulting from attenuated HigB expression in *P*. *aeruginosa*.

Since T3SS plays a crucial role in the interaction between *P*. *aeruginosa* and the host immune system, particularly in immune evasion and cytotoxicity towards host cells [41,42], we subsequently assessed and compared the expression of key regulators and effectors of T3SS in WT and mutant strains under LL-37 stimulation (Fig 6E). We demonstrated that LL-37 treatment significantly upregulated the expression of *exsA* and effectors *exoS*, *exoT*, and *exoU* by 2.74- to 6.68-fold in the WT strain compared to untreated group. In contrast, the combined mutations of CprRS components and *higB* gene greatly attenuated the upregulation of these T3SS proteins expression in response to LL-37 stimulation. In bacterial cytotoxicity assays, LL-37-treated WT strains exhibited increased cytotoxicity towards Raw264.7 macrophages compared to the untreated group (Figs 6F and S7). However, the mutant strains all showed significantly reduced cytotoxicity relative to their corresponding WT counterparts. These findings further support the essential role of HigB-mediated T3SS secretion activation, regulated by CprRS, in determining the full bacterial cytotoxicity.

### CprRS deficiency aggravated inflammatory responses and attenuated virulence in *P*. *aeruginosa* infection

**Fig 7:**
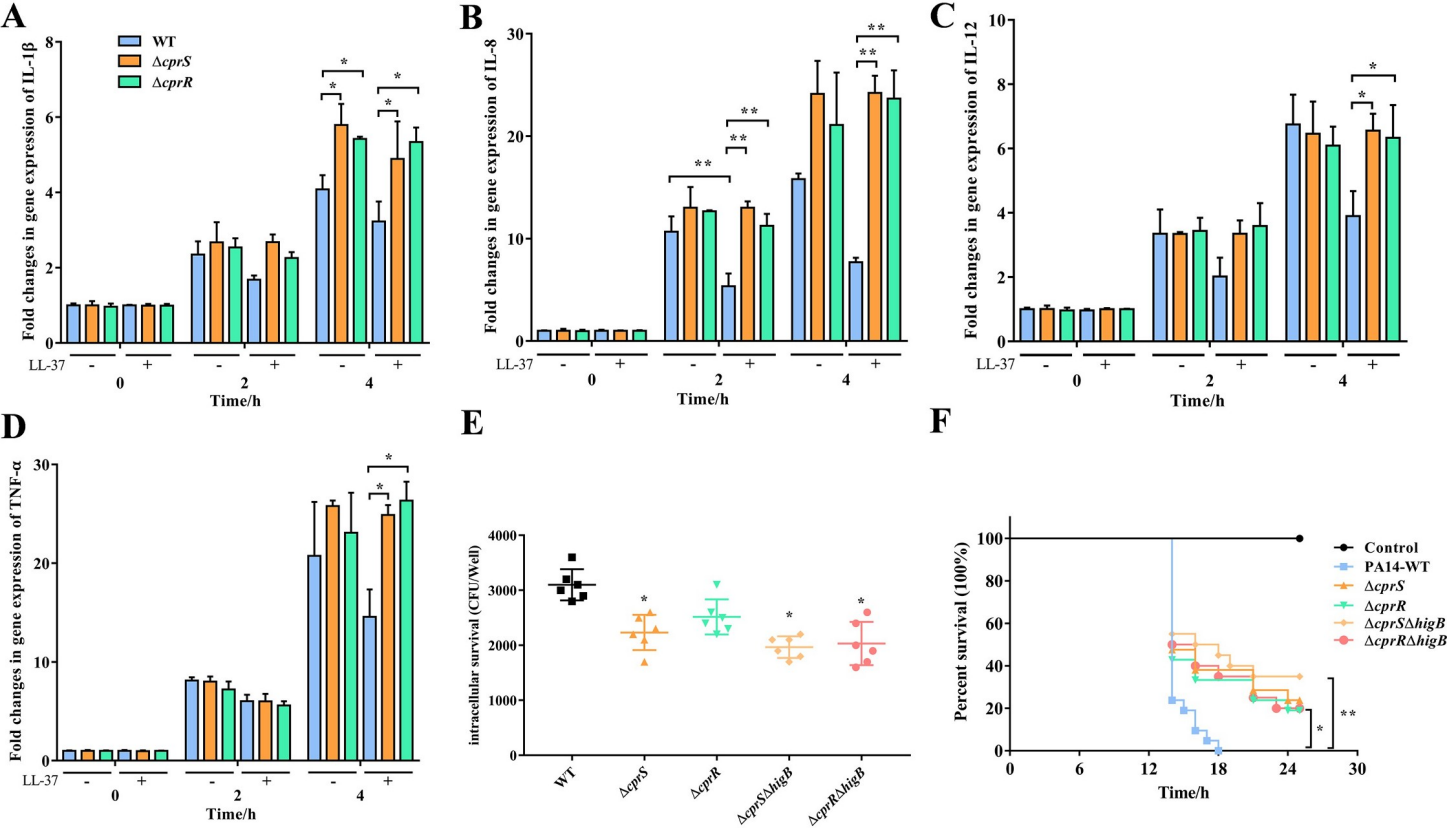
Effect of *cprRS* deletion on modulating of host immune response and invasion ability. (A) to (D) The levels of intracellular cytokines from Raw264.7 cells infected with WT or mutants. The *18s RNA* gene was used as a normalizer. (E) Survival assay of WT and mutants in RAW264.7 macrophages. Sterile PBS served as negative control. The RAW264.7 macrophages were seeded at 2 × 10^5^ density/well on a 24-well plate and cultured in DMEM for 16–18 h. The cells were infected with different *P*. *aeruginosa* strains at a MOI of 10 for 24 h, then the cells were washed with sterile PBS and lysed for serial dilution and plating on LB agar plates. All experiments were performed in triplicates and error bars represent error of the mean (±) for SD. *P <0.05; **P < 0.01; ***P < 0.001 by one-way ANOVA statistical test. (F) Measurement virulence of the WT and mutant strains in a *G*. *mellonella* infection model. The method is same with Fig 1E.

Generally, LL-37 possesses crucial antimicrobial properties in humans, primarily attributed to its anti-inflammatory activity and selective modulation of favorable immune responses against various pathogens [43,44]. In the case of *P*. *aeruginosa* infections, the bacteria heavily rely on the T3SS to deliver effectors into host cells, thereby interfering with the expression of pro-inflammatory factors and subverting host immunity [28,41]. To explore the impact of CprRS on host inflammatory responses, we assessed the levels of critical inflammatory cytokines [tumor necrosis factor–α (TNF-α), interleukin-1β (IL-1β), IL-8, and IL-12] in RAW264.7 macrophages following infections [45]. Remarkably, we observed significantly elevated levels of these pro-inflammatory cytokines in cells infected with Δ*cprS* and Δ*cprR* strains compared to the WT strain (Fig 7A–7D), indicating that deletion of *cprRS* intensified the inflammatory response. Furthermore, pretreatment with LL-37 enhanced the ability of the WT strain to effectively inhibit the activation of pro-inflammatory cytokines, a response that was largely absent in Δ*cprS* and Δ*cprR* strains (Fig 7A–7D).

To further elucidate the effects associated with the attenuated phenotype of the mutant strains, we conducted macrophage invasion assays to examine whether the CprRS system contributes to intracellular survival. The results revealed that both *cprRS* and *higB* deletions led to a 1.6-fold reduction in the number of intracellular bacteria compared to the WT strain after 24 h of infection (Fig 7E). In the *G*. *mellonella* infection model, loss of *cprRS* and *higB* significantly decreased larval mortality compared to the WT strain, with Δ*cprS*Δ*higB* causing larval death to decrease by 61.2% within 24 h (Fig 7F). Conversely, larva infected with the WT strain and Δ*cprR* exhibited higher mortality rates, reaching 100% and 81.9% by 24 h, respectively. Collectively, these findings indicate that the CprRS system plays a pivotal role in recognizing host LL-37 and subsequently regulating virulence.

## Discussion

**Fig 8:**
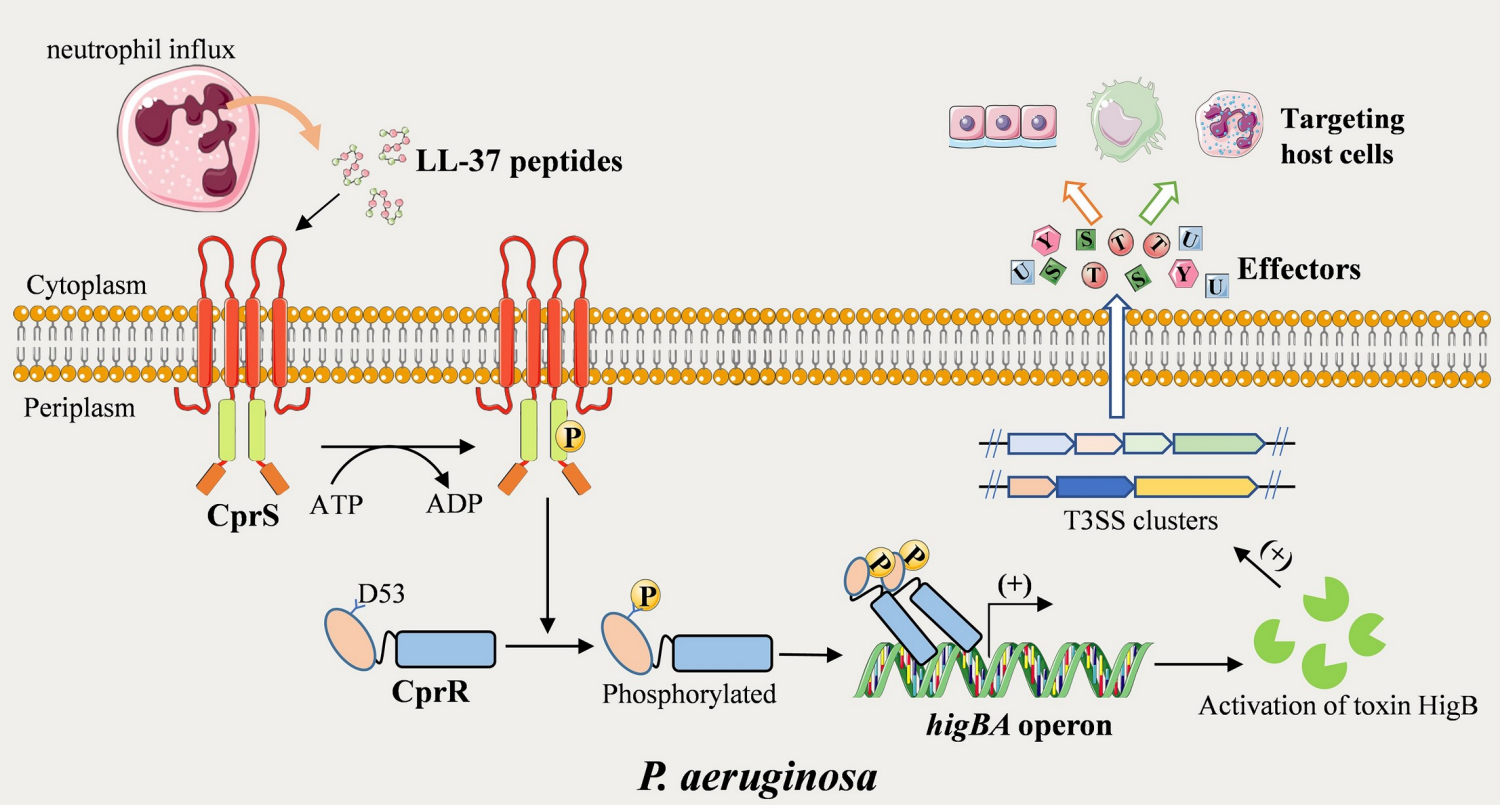
Proposed model of CprRS sensing the host LL-37 to regulate expression of T3SS in *P*. *aeruginosa*. In early stage of infection, the host neutrophils produce excessive LL-37 peptides to destroy invading bacteria. The CprS in *P*. *aeruginosa* senses the external LL-37 and phosphorylates the cognate regulator CprR at position D53. CprR recognizes the palindrome sequence in the promoter region of the *higBA* to enhance the expression levels of toxin HigB, leading to the activation of T3SS to modulate host immune response.

TCSs represent the most common signal-transduction cascades in bacteria, sensing external cues such as ions, peptides, and compounds [46,47]. Canonical TCSs comprise a transmembrane sensor histidine kinase (HK) and an intracellular response regulator (RR) [47]. Upon binding to specific stimuli, the HK undergoes autophosphorylation and transfers the phosphoryl group to the RR, activating its transcription regulation ability to influence bacterial behavior. To date, over 60 TCSs have been identified in the *P*. *aeruginosa* PAO1 genome, they have been recognized as crucial players in various biological processes such as ion homeostasis, drug resistance, metabolic regulation, pili synthesis, and pathogenesis [15,19]. In particular, many TCSs have been identified in *P*. *aeruginosa*, and substantial evidence suggests their involvement in the regulation of virulence factors [14]. For instance, the GacSA network responds to c-di-GMP levels, positively controlling the production of the autoinducer N-butyryl-homoserine lactone and the formation of virulence factors, including pyocyanin and T3SS [48]. The CbrAB system recognizes different carbon sources and plays a positive role in T3SS regulation and effector release [49]. The calcium-responsive kinase LadS collaborates with other TCSs to modulate T3SS activity and specifically regulates the expression of ExoU, serving as an acute-to-chronic virulence switch [50]. The CprRS TCS was initially identified in *P*. *aeruginosa* PAO1 strain H103 for adaptive resistance to cationic peptide antibiotics, including Polymyxin B and indolicidin [26]. We found that the sensor domain of CprS directly sensed the human host defense peptide LL-37 and phosphorylated the cognate regulator CprR at position D53. Further research showed that CprR enhance the expression levels of toxin HigB. On the other hand, the *higB* gene from the type II TA system *higBA* has been extensively studied and shown to be closely associated with T3SS regulation [35], biofilm formation [33], iron uptake [34], and persistence [35]. The HigBA system consists of an endonuclease-type toxin, HigB, and its cognate antitoxin, HigA. During normal conditions, HigA recognizes the pseudopalindromic sequence (5’-TTAAC GTTAA-3’) in the promoter region of *higB* to repress its expression [31]. However, under stress conditions such as antibiotic treatment, HigA is rapidly degraded by proteases, resulting in the overproduction of HigB, leading to increased T3SS activity and the formation of persister cells. Based on these findings, we propose a plausible model in which CprRS plays an essential role in regulating bacterial virulence by sensing LL-37 from the host immune system (Fig 8). These findings underscore the importance of understanding the extensive TCS network in *P*. *aeruginosa* and shed light on the interplay between host immune signals and bacterial virulence regulation.

In the field of microbiology and immunology, the interaction between host antimicrobial peptides and pathogenic bacteria has been a subject of extensive research [2,6]. The success of bacterial infections depends on a delicate balance of signals exchanged between microbes and hosts. Cationic antimicrobial peptides, including defensins such as α-defensins, β-defensins, and cathelicidin LL-37, are crucial components of innate host defense against bacterial infections [51]. Numerous studies have shown that LL-37 acts as a shield between the host and the external environment, preventing bacterial infections, and the loss of LL-37 leads to severe bacterial skin infections in animal models [52]. While several TCSs have been reported to be activated by LL-37, such as the virulence sensor kinase CovS in group A *streptococcus* and the CarSR system in *Vibrio cholerae*, which sense human enteric α-defensin 5 and LL-37 to promote bacterial pathogenicity [53], little is known about whether TCSs in *P*. *aeruginosa* can serve as receptors for LL-37 and impact bacterial virulence. In our study, we demonstrated that the CprRS system, a highly conserved TCS in *P*. *aeruginosa*, specifically recognized LL-37 and modulated T3SS activities, which are critical for causing cytotoxicity and evading host immune responses. This sheds light on how *P*. *aeruginosa* coordinates T3SS expression during various stages of infection. Recent evidence suggests that alginate production in *P*. *aeruginosa* may modify the innate immune response by increasing resistance to phagocytosis and promoting pulmonary persistence [54]. In the presence of LL-37, the *cprS* mutant notably upregulated the negative regulator MucA and downregulated the key protease AlgW, both of which are part of the intramembrane proteolysis (RIP) cascade for alginate synthesis [40]. These findings indicate the potential involvement of the CprRS system in mediating additional virulence pathways to counteract the human immune system. Consistent with previous studies, we also showed that the CprRS system significantly contributed to polymyxin resistance by inducing the expression of the *arn* operon under LL-37 conditions. Moreover, it is noteworthy that the model insect *G*. *mellonella* possesses at least 18 antimicrobial peptides (AMPs), a substantial number of which belong to the cationic category, such as Cecropin A and Peptide 5.11.1 [55,56]. Interestingly, Cecropin A, a 37 amino acid peptide, has been found to disrupt biofilms of uropathogenic *E*. *coli*, suggesting its potential similarity to human LL-37 [57]. These cationic AMPs in *G*. *mellonella* mimic the environment in which bacteria infect human lungs, which could have significant implications for the virulence of mutants as compared to the WT strain. Whether the CprRS system recognizes different cationic AMP signals and how it does so remain a subject for future research. Polymyxin antibiotics are commonly used as a last line of defense against multidrug-resistant Gram-negative bacteria in clinical medicine [58], but the emergence of antibiotic-resistant strains poses a serious threat. Encouragingly, the *cprS* mutant exhibited reduced Polymyxin resistance, suggesting a new drug target that could extend the useful lifespan of antibiotics.

T3SS is a complex "molecular syringe" that injects various effectors (e.g., ExoS, ExoU, ExoY, and ExoT) that target essential cell signaling pathways [28,41], disrupt cytoskeleton synthesis, and inhibit inflammasome activation. T3SS also activates caspase-1 proteolysis and inflammasome-mediated responses, resulting in pyroptosis and the release of inflammatory cytokines. Inflammasome activation has been implicated in *P*. *aeruginosa* pathogenicity, affecting bacterial clearance. We previously discovered that the type II TA system HigBA was induced by antibiotic treatments and positively regulated the expression of the T3SS [32]. However, the expression of type II TA systems is tightly autoregulated, the mechanisms about how bacteria sense external cues and mediate TA system activity remained elusive. In this study, both in vitro and in vivo studies demonstrated that CprR activates *higB* expression by binding to the palindrome sequence in the *higBA* promoter region, leading to increased levels of T3SS proteins. These critical findings reveal that type II TA systems can be regulated by other specific regulators, allowing for more flexibility in modulating diverse bacterial metabolisms.

In conclusion, our study unveils that CprS is required for virulence regulation and Polymyxin resistance in *P*. *aeruginosa*. This highlights that CprS serve as a novel host-sensing protein in *P*. *aeruginosa* that activates CprR and downstream T3SS genes to adapt to outside-host environment. These findings promote a better understanding of the interkingdom communication between the host immune signals sensed by bacteria and the subsequent releases of bacterial virulence factors. Future investigations should focus on elucidating the mechanisms of CprS interaction with LL-37 and identifying key residues involved in recognition. Given the essential roles of the CprRS system, this highly conserved system holds promise as a drug target for *P*. *aeruginosa* therapy. Moreover, the high affinity of CprS for human LL-37 could be explored for LL-37 detection and the early diagnosis of inflammatory responses.

## Materials and methods

### Bacterial strains, media, and growth conditions

Bacteria strains and plasmids used in this work are listed in S4 Table. All mutants were constructed by gene allelic substitution as described in methods. Both *E*. *coli* and *P*. *aeruginosa* culturing was performed using LB medium at 37°C with shaking (220 r/min). For plasmid maintenance, antibiotics were used at the following concentrations: for *P*. *aeruginosa*, gentamicin at 50 μg/mL in LB medium, tetracycline at 120 μg/mL, 13 μg/mL LL-37, and/or 25 μg/mL irgasan in LB medium, for *E*. *coli*, ampicillin at 50 μg/mL, kanamycin at 50 μg/mL, and gentamicin at 15 μg/mL.

### Construction of *P*. *aeruginosa* mutant strains

Briefly, two-step allelic exchange method was used to construct *P*. *aeruginosa* mutant strains [34]. The upstream (600–800 bp) and downstream (600–800 bp) fragments of *cprR*, *cprS* and *higB* genes from the *P*. *aeruginosa* PA14 were subcloned into the suicide plasmid pEX18Gm by using One Step Cloning Kit. The vectors were first transformed into *E*. *coli* S17-1 and mobilized into *P*. *aeruginosa* PA14 through incubating with S17-1. The colonies were firstly screened in LB agar containing 50 μg/mL gentamicin and then 10% sucrose agar, then identified by PCR. All the primers used in this work are listed in S5 Table.

### *G*. *mellonella* killing assays

The different *P*. *aeruginosa* strains were grown in LB media to an optical density at OD_600_ reached to 0.5–0.6, then collected and washed 3 times with sterile PBS. Each *G*. *mellonella* was injected with 10 μL *P*. *aeruginosa* dilution (5 × 10^3^ CFU/mL, serial dilution, and plate counts), the *G*. *mellonella* injected with sterile PBS as control. The *G*. *mellonella* were then stationary incubated at 37°C and monitored in next 24 h.

### Total protein extraction and bicinchoninic acid (BCA) protein assay

Protein was collected, reduced, and alkylated from WT, Δ*cprS*, Δ*cprR*, Δ*cprS*Δ*higB*, and Δ*cprS*Δ*higB* strains as reported [32]. The total purified peptides were solubilized in 20 μL of 0.1% formic acid (FA, v/v) and the concentration was measured according to BCA assay.

### Mass spectrometry analysis

About 2 μg samples were analyzed by orbitrap Fusion Lumos MS coupled online to an EASY-nLC 1200 system with a data-independent acquisition mode (DIA). The mobile phase used for the liquid chromatography consisted of buffer A (0.1% FA) and buffer B (80% acetonitrile, 0.1% FA). The peptides were separated using a 160-min nonlinear gradient consisting of 4–35% buffer B for 85 min, 35–100% buffer B for 60 min and 100% buffer B for 15 min at a flow rate of 300 nL min^−1^. The acquired DIA data were analyzed by DIA-NN software against the protein database of *P*. *aeruginosa* PA14. Data processing was conducted using the software Perseus. Student’s *t*-test was used to assess the significance of differential expression of proteins (DEPs) between two groups. Proteins that have significance level of *P* < 0.05 and fold change >1.5 or <–1.5 were considered as DEPs.

### Protein expression and purification

The *E*. *coli* BL21 (DE3) strains harboring pET22b-*cprS* and pET22b-*cprR* were cultured at 37°C in LB medium and suppled with 0.4 mM IPTG as the OD_600_ reached to 0.8. The cultures were incubated at 16°C for an additional 14 h and the cells were collected and resuspended in buffer A (20 mM tris-HCl (pH 8.0), 150 mM NaCl). The cells were lysed by ultrasound and centrifuged at 15, 000 rpm for 20 min at 4°C. Then the supernatant was applied to Ni-agarose resins and eluted with buffer containing 300 mM imidazole. As for full-length CprS, the supernatant needed to be extracted by 2% ELUGENT Detergent and flow 0.02% DDM for elution. The target proteins were further purified via AKTA purifier using buffer A and concentrated to 12 mg/mL.

### Microscale thermophoresis for peptide binding assay

The 100 nM CprS with His-tag was labeled with Monolith His-Tag Labelling Kit RED-tris-NTA 2nd Generation Kit and the cationic peptides used in this work were diluted with PBST buffer gradient from 0.5 mM [34]. The proteins and cationic peptide dilutions were mixed and incubated at 25°C for 30 min and detected on a Monolith NT.115 instrument. The data were analyzed by NanoTemper Analysis 1.2.20 software.

### Electrophoretic mobility shift assays

The gene promoters were obtained by PCR. The CprR and DNA fragment were mixed in different ratios (total 10 μL buffer, 25 mM Tris–HCl pH 7.5, 150 mM NaCl, 1 mM MgCl_2_, 2 mM KCl) and incubated at 4°C for 30 min, then the samples were analyzed by 8% polyacrylamide gel electrophoresis in 0.5 × TBE (Tris/boric acid/EDTA) buffer at 130 V for 120 min. The bands were staining with EB and visualized through BioRad imager.

### RNA extraction and real-time quantitative PCR (RT-PCR)

Overnight bacterial cultures were subcultured in fresh LB medium to OD_600_ 0.8 and the cells were collected by using Trizol. The cDNA was synthesized by using PrimeScript RT reagent Kit. 2 × SYBR qPCR Master Mix was used to perform qPCR assays [34]. The reaction step was 2 × SYBR qPCR Master Mix (Without ROX) 10 μL, upstream Primer (10 μM) 0.4 μL, downstream Primer (10 μM) 0.4 μL, template cDNA 1 μg and ddH_2_O up to 20 μL. The qPCR procedure was set as: stage 1 (95°C 30 s, repeat once), stage 2 (95°C 10 s, 60°C 30 s, repeat for 40 times) and stage 3 (95°C 15 s, 60°C 1 min, 95°C 15 s, repeat 1 time). The *oprL* gene was used as a normalizer. The same method was used for mammalian cells, and *18 sRNA* served as a normalizer.

### MIC determination

The initial concentrations of meropenem, cephalosporin, tetracycline, gentamicin, ciprofloxacin, polymyxin B, and colistin are: 0.1 mg/mL, 0.02 mg/mL, 0.5 mg/mL, 0.16 mg/mL, 1 mg/mL, 0.1 mg/mL, and 0.1 mg/mL, all the antibiotics were 10 × gradient dilution with sterile PBS buffer. Overnight bacterial cultures of WT *P*. *aeruginosa* and mutants were re-inoculated into fresh LB medium supplemented at OD_600_ = 0.1, then 90 μL cultures and 10 μL antibiotic dilutions were added in a 96-well microtiter dish for 24 h at 37°C. The MIC values was determined by plating on LB agar plates for 16 h at 37°C.

### β-Galactosidase assay

The 300 bp promoter region of *higB* was cloned upstream of a promoterless *lacZ* gene in pRG970km to construct *lacZ*-report plasmid. The vector pRG970km and pET22b-*cprR* were co-transformed into *E*. *coli* BL21 (DE3) and screened in LB agar media containing 50 μg/mL kanamycin and ampicillin at 50 μg/mL. The bacteria were grown in LB medium as OD_600_ reached to 0.6 at 37°C, then added with 0.1 mM IPTG for another 3 h. The cells were collected and the galactosidase activities were measured as previously reported. β-Galactosidase activity was normalized by the galactosidase activity at the time of induction.

### Cytotoxicity assays

The Raw264.7 cells were infected with suspended bacteria at a multiplicity of infection (MOI) of 10 in antibiotic-free DMEM medium. Following incubation, the supernatant was collected and the presence of LDH was measured using the LDH cytotoxicity assay kit [35]. The baseline level of LDH release (0% LDH release) was determined using DMEM medium. Cells treated with LDH release agent C0017-1 were used as a positive control for total release (100% LDH release). The percentage of cytotoxicity was calculated according to the instructions provided by the manufacturer.

### Macrophage uptake and intracellular survival

Intracellular survival assay was performed as previously reported [35]. In brief, the RAW264.7 macrophages were seeded at 2 × 10^5^ density/well on a 24-well plate and cultured in DMEM for 16–18 h. The cells were infected with different *P*. *aeruginosa* strains at a MOI of 10 for 24 h, then the cells were washed with sterile PBS and lysed for serial dilution and plating on LB agar plates.

### Statistics

The data were analyzed using GraphPad Prism 7.0 software and exhibited as the mean ± the standard error of the mean (SEM). Experiments were performed using a minimum of three biologically distinct replicates. For comparisons of two groups, including mRNA levels and LDH activities, Student’s *t* test (two-tailed) was used. β-Galactosidase assay and intracellular survival were analyzed by one-way ANOVA with Bonferroni correction for multiple comparisons. For studies with *G*. *mellonella* infection assays, Mantel-Cox test for statistics was performed. Significant differences were determined at *P* < 0.05.

## Supporting information

S1 Fig Conserved residues of CprR for phosphorylated modification by CprS. (A) Part of sequence alignment on CprR and other homologous proteins, including *Thermotoga maritima* PhoB, *Mycobacterium tuberculosis* H37Rv Regx3 and PhoP, *Escherichia coli* str. K-12 substr. MG1655 BaeR, and *Klebsiella pneumoniae* JM45 PmrA. The conserved Aspartate residue is labeled with solid asterisk. (B) CprS and CprR were purified with Ni-NTA resin and further purified with size exclusion chromatography. The reaction mixtures containing 25 mM Tris-HCl pH8.0, 150 mM NaCl, 15 mM MgCl_2_, 50 mM DTT, 5 mM ATP, 0.1 μM CprS and 5 μM CprR were treated at 25°C, then electrophoresed on 10% native-PAGE gel.
(TIF)

S2 Fig CprS contributes to virulence and Polymyxin B resistance in *P*. *aeruginosa*. (A) Hierarchical clustering of the z-scored extracted ion chromatogram (left panel) was used to evaluate the reproducibility of the proteome quantification in WT and Δ*cprS* strains under LL-37 treatment, the significant expressed proteins are categorized by functional category (right panel). (B) Hierarchical clustering of the zscored extracted ion chromatogram (left panel) was used to evaluate the reproducibility of the proteome quantification in Δ*cprS* strains before and after LL-37 treatment, the significant expressed proteins are categorized by functional category (right panel). (C) Polymyxin B MICs of WT and mutants in LB medium.
(TIF)

S3 Fig CprR directly interacts with *higB* promoter. (A) The binding affinity of CprR toward *higB* promoter was measured with MST. The CprR was pretreated with AcP and then labeled with Monolith His-Tag Labelling Kit RED-tris-NTA 2nd Generation Kit. The final protein concentration was 100 nM and DNA fragments have 16 doubling dilutions started from 10 μM (B) EMSAs showing that native CprR rather than mutant CprR could bind to the promoter region of *bkdA1*. Each reaction mixture contains PCR products (1 μM) and the CprR protein concentrations were indicated above the lane.
(TIF)

S4 Fig HigBA system is activated in the presence of LL-37. (A) β-galactosidase reporter system to determine the transcription activity of *higB* promoter in *P*. *aeruginosa*. (B) The mRNA levels of *higA* in mutants compared WT. Error bars indicate the means ± SD of three independent experiments. *P <0.05; **P < 0.01; ***P < 0.001. (C) β-galactosidase reporter system to determine the transcription activity of *higB* and *higA* in *P*. *aeruginosa* under LL-37 treatment. *higA* mRNA could be expressed separately from a promoter inside *higB* (marked as P2). (D) Degradation of HigA under LL-37 treatment. PAO1 expressing HigA-His_6_ were treated with LL-37 as (C) and analyzed by Western blotting, and the anti-RNA polymerase beta (RNAP) antibody was used as a negative control to determine the level of HigA.
(TIF)

S5 Fig Overall structure of CprR obtained from Alphafold. (A) Ribbon representation of the CprR. The overall structure of CprR comprises 8 α-helixes and 10 β-sheets. (B) Part of sequence alignment on CprR and other homologous proteins with known structures, including *Thermotoga maritima* PhoB, *Mycobacterium tuberculosis* H37Rv Regx3 and PhoP. The potential residues involved in DNA interaction are labeled with solid asterisk.
(TIF)

S6 Fig Heatmap showing the protein changes of WT and mutants under LL-37 treatment. Hierarchical clustering of the z-scored extracted ion chromatogram was used to evaluate the reproducibility of the proteome quantification in WT, Δ*cprS*Δ*higB*, and Δ*cprR*Δ*higB* strains under LL-37 treatment.
(TIF)

S7 Fig CprRS system contribute to cytotoxicity towards host cells. Raw264.7 cell were infected by different strains at an MOI of 10 for 4 h, the relative cytotoxicity was then determined by the LDH release assay. Error bars indicate the means ± SD of three independent experiments. *P <0.05; **P < 0.01; ***P < 0.001.
(TIF)

S1 Table List of genes with potential CprR-binding site in promoter regions. (DOCX)

S2 Table Significant downregulated proteins in Δ*cprR*Δ*higB* compared with WT after LL-37 treatment. (DOCX)

S3 Table Significant downregulated proteins in Δ*cprS*Δ*higB* compared with WT after LL-37 treatment. (DOCX)

S4 Table Bacteria strains and plasmids. (DOCX)

S5 Table Primers used in the work. (DOCX)
