# Supplementary figures and images for: Pseudomonas aeruginosa two-component system CprRS regulates HigBA expression and bacterial cytotoxicity in response to LL-37 stress
Source: PLoS Pathog. 2024 Jan 10;20(1):e1011946. doi: 10.1371/journal.ppat.1011946 (PMC10805311; doi:10.1371/journal.ppat.1011946)

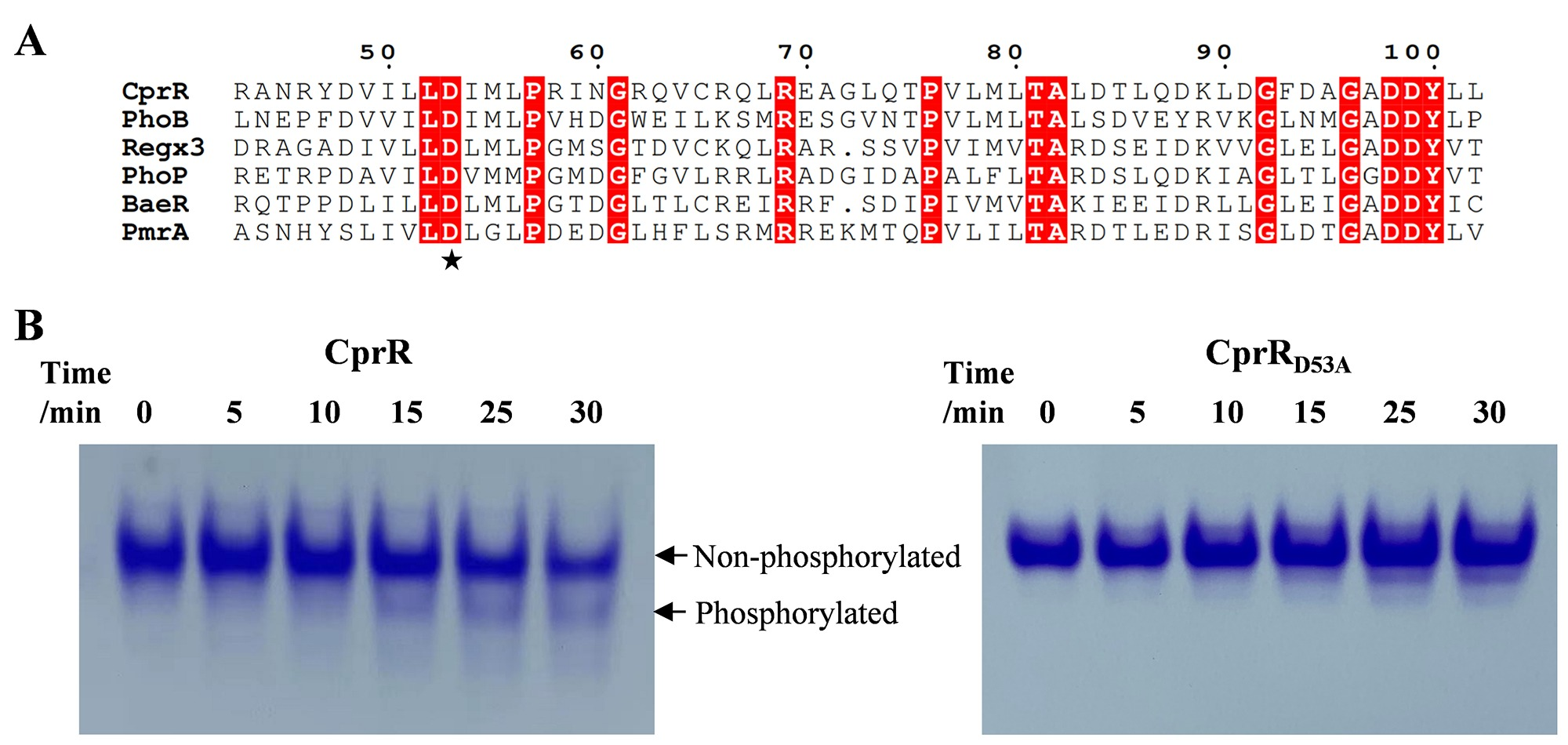

Supplement: S1 Fig — (A) Part of sequence alignment on CprR and other homologous proteins, including Thermotoga maritima PhoB, Mycobacterium tuberculosis H37Rv Regx3 and PhoP, Escherichia coli str. K-12 substr. MG1655 BaeR, and Klebsiella pneumoniae JM45 PmrA. The conserved Aspartate residue is labeled with solid asterisk. (B) CprS and CprR were purified with Ni-NTA resin and further purified with size exclusion chromatography. The reaction mixtures containing 25 mM Tris-HCl pH8.0, 150 mM NaCl, 15 mM MgCl2, 50 mM DTT, 5 mM ATP, 0.1 μM CprS and 5 μM CprR were treated at 25°C, then electrophoresed on 10% native-PAGE gel. (TIF) [file ppat.1011946.s001.tif]

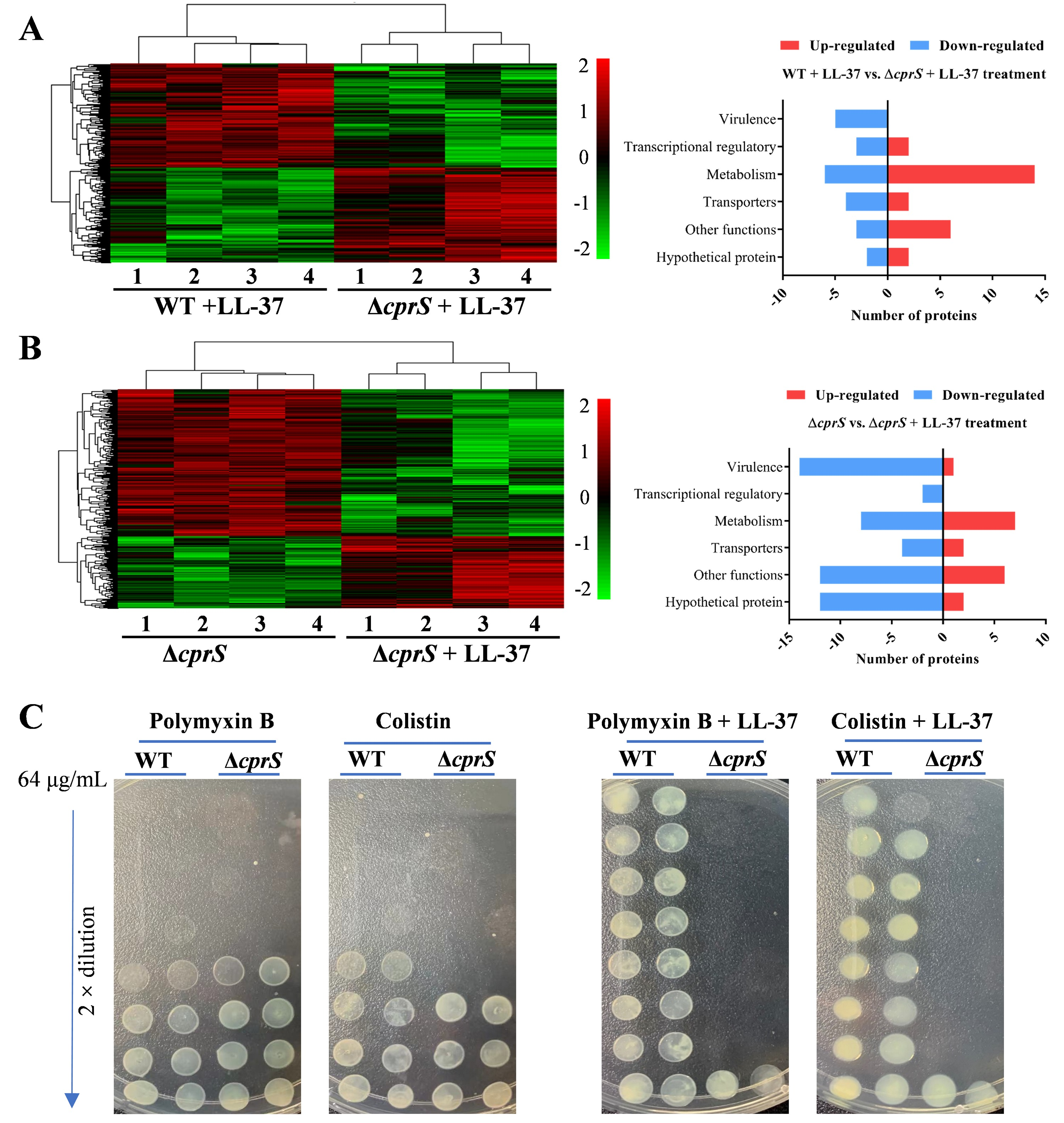

Supplement: S2 Fig — (A) Hierarchical clustering of the z-scored extracted ion chromatogram (left panel) was used to evaluate the reproducibility of the proteome quantification in WT and ΔcprS strains under LL-37 treatment, the significant expressed proteins are categorized by functional category (right panel). (B) Hierarchical clustering of the zscored extracted ion chromatogram (left panel) was used to evaluate the reproducibility of the proteome quantification in ΔcprS strains before and after LL-37 treatment, the significant expressed proteins are categorized by functional category (right panel). (C) Polymyxin B MICs of WT and mutants in LB medium. (TIF) [file ppat.1011946.s002.tif]

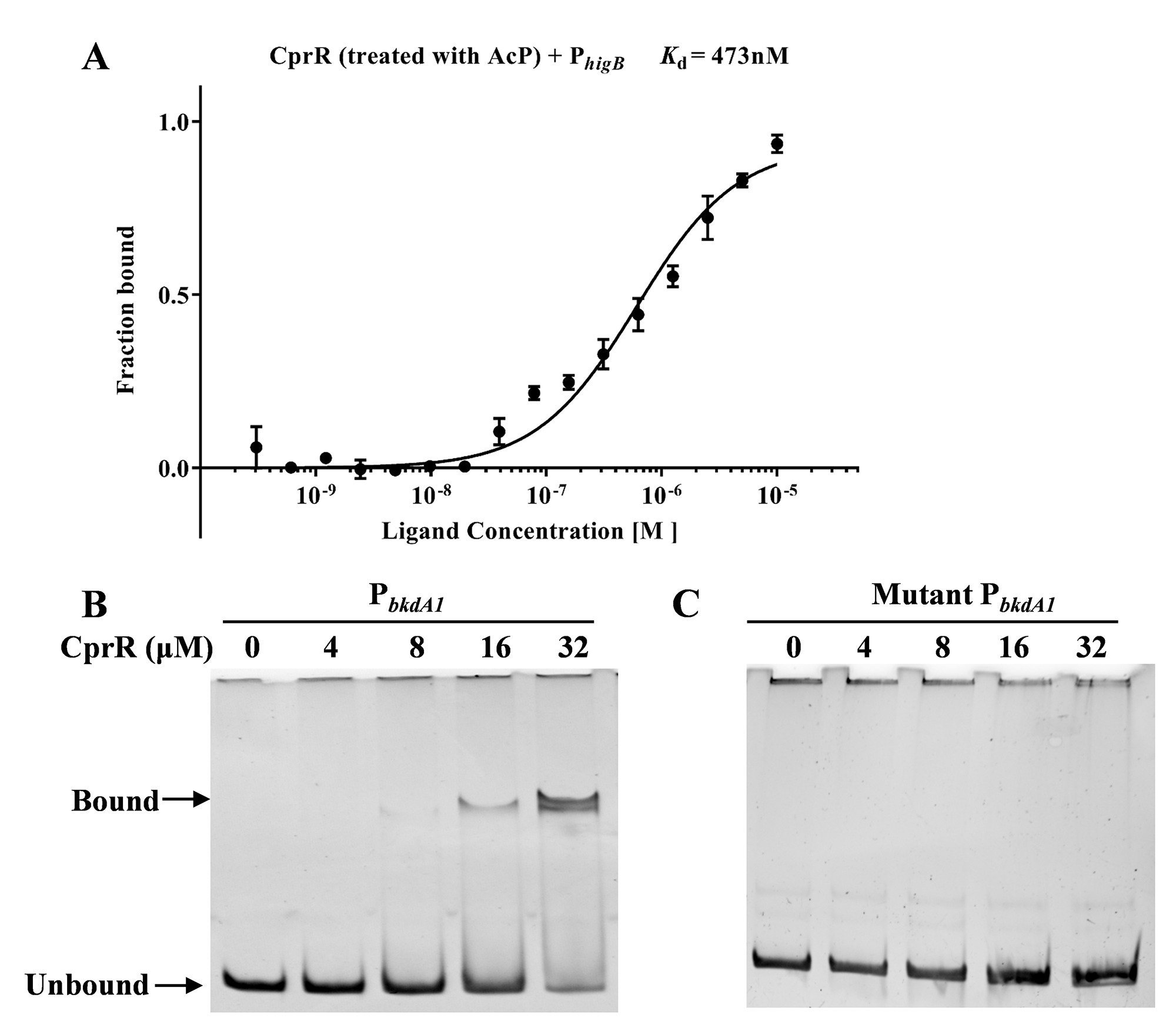

Supplement: S3 Fig — (A) The binding affinity of CprR toward higB promoter was measured with MST. The CprR was pretreated with AcP and then labeled with Monolith His-Tag Labelling Kit RED-tris-NTA 2nd Generation Kit. The final protein concentration was 100 nM and DNA fragments have 16 doubling dilutions started from 10 μM (B) EMSAs showing that native CprR rather than mutant CprR could bind to the promoter region of bkdA1. Each reaction mixture contains PCR products (1 μM) and the CprR protein concentrations were indicated above the lane. (TIF) [file ppat.1011946.s003.tif]

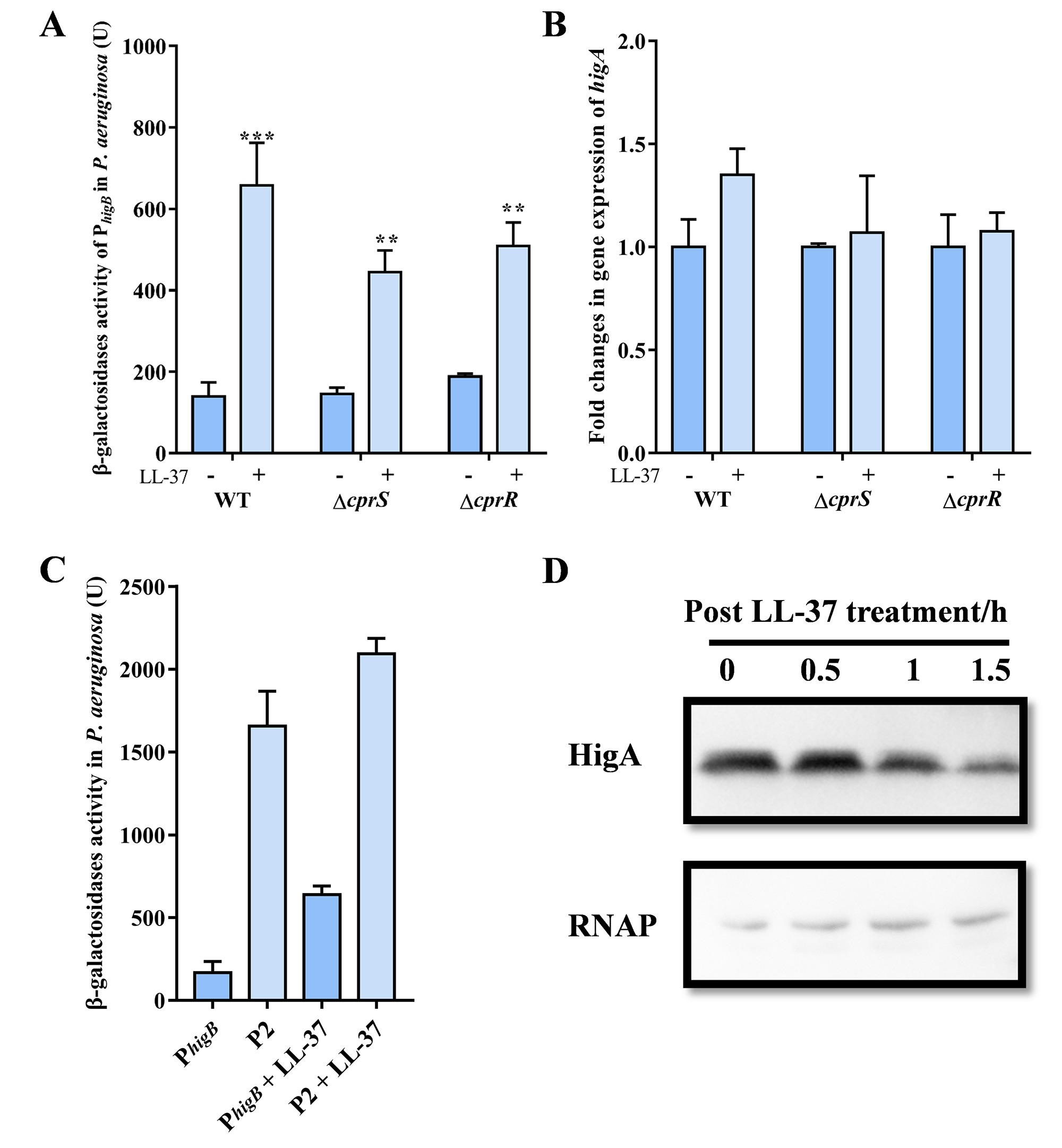

Supplement: S4 Fig — (A) β-galactosidase reporter system to determine the transcription activity of higB promoter in P. aeruginosa. (B) The mRNA levels of higA in mutants compared WT. Error bars indicate the means ± SD of three independent experiments. *P <0.05; **P < 0.01; ***P < 0.001. (C) β-galactosidase reporter system to determine the transcription activity of higB and higA in P. aeruginosa under LL-37 treatment. higA mRNA could be expressed separately from a promoter inside higB (marked as P2). (D) Degradation of HigA under LL-37 treatment. PAO1 expressing HigA-His6 were treated with LL-37 as (C) and analyzed by Western blotting, and the anti-RNA polymerase beta (RNAP) antibody was used as a negative control to determine the level of HigA. (TIF) [file ppat.1011946.s004.tif]

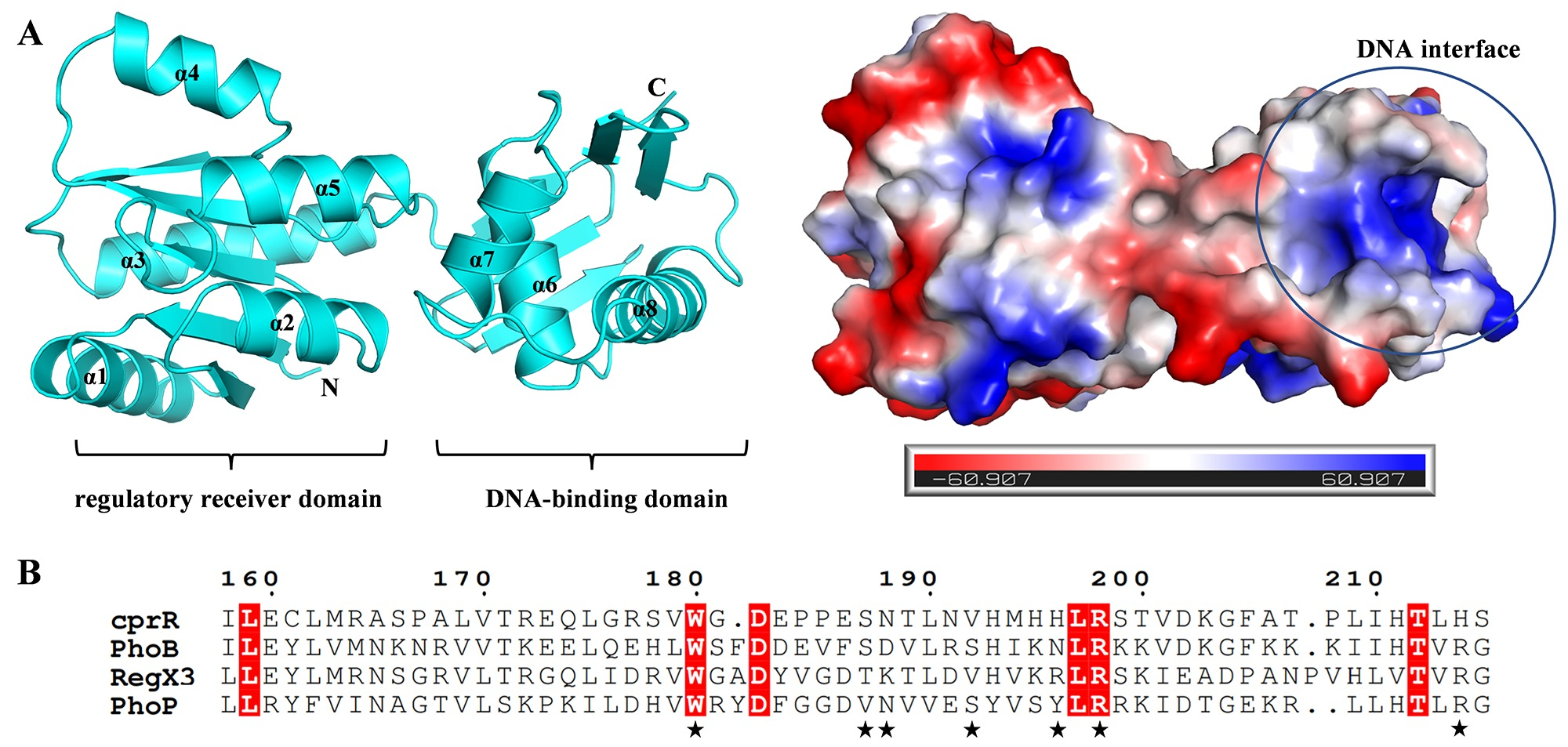

Supplement: S5 Fig — (A) Ribbon representation of the CprR. The overall structure of CprR comprises 8 α-helixes and 10 β-sheets. (B) Part of sequence alignment on CprR and other homologous proteins with known structures, including Thermotoga maritima PhoB, Mycobacterium tuberculosis H37Rv Regx3 and PhoP. The potential residues involved in DNA interaction are labeled with solid asterisk. (TIF) [file ppat.1011946.s005.tif]

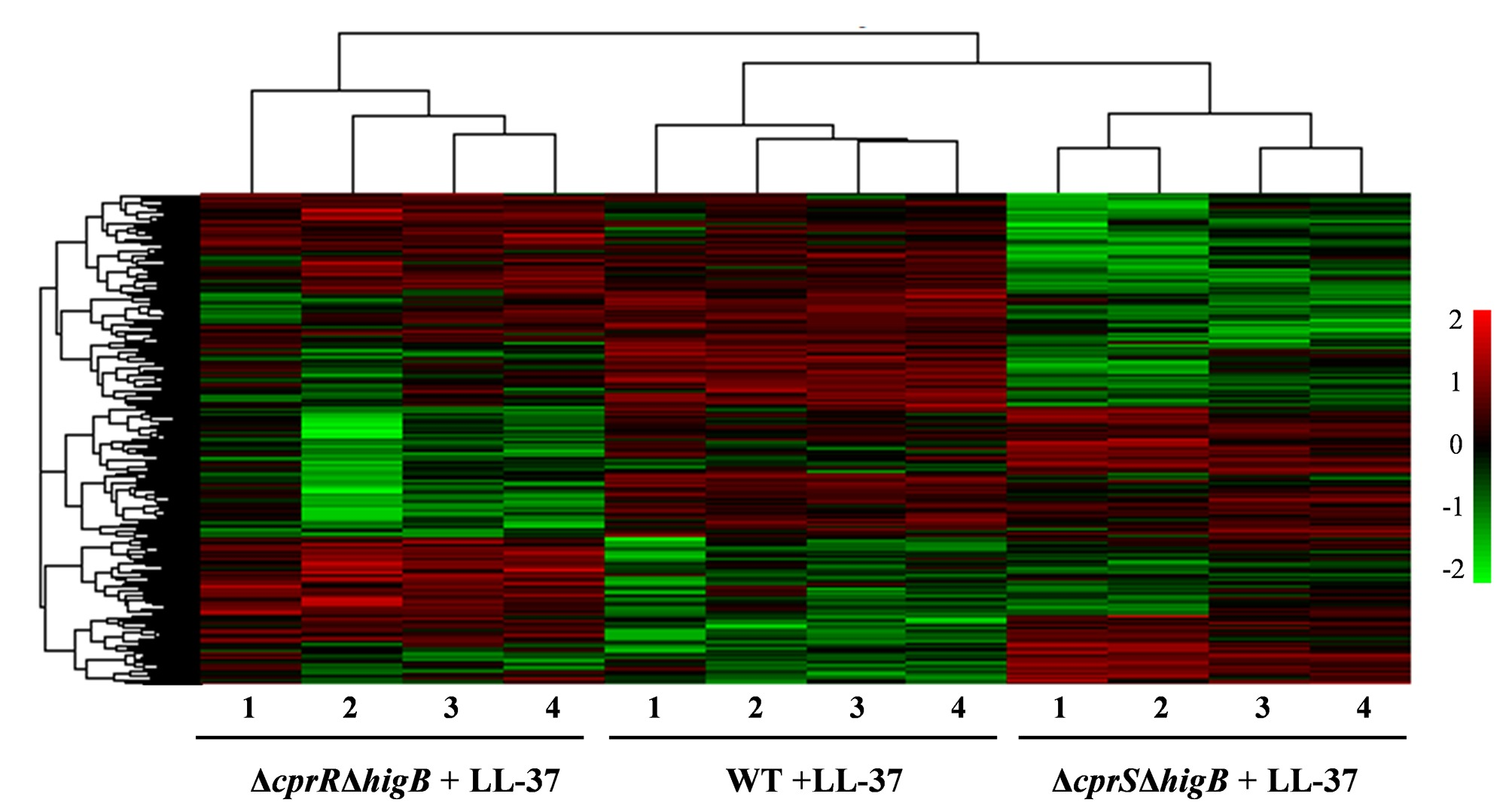

Supplement: S6 Fig — Hierarchical clustering of the z-scored extracted ion chromatogram was used to evaluate the reproducibility of the proteome quantification in WT, ΔcprSΔhigB, and ΔcprRΔhigB strains under LL-37 treatment. (TIF) [file ppat.1011946.s006.tif]

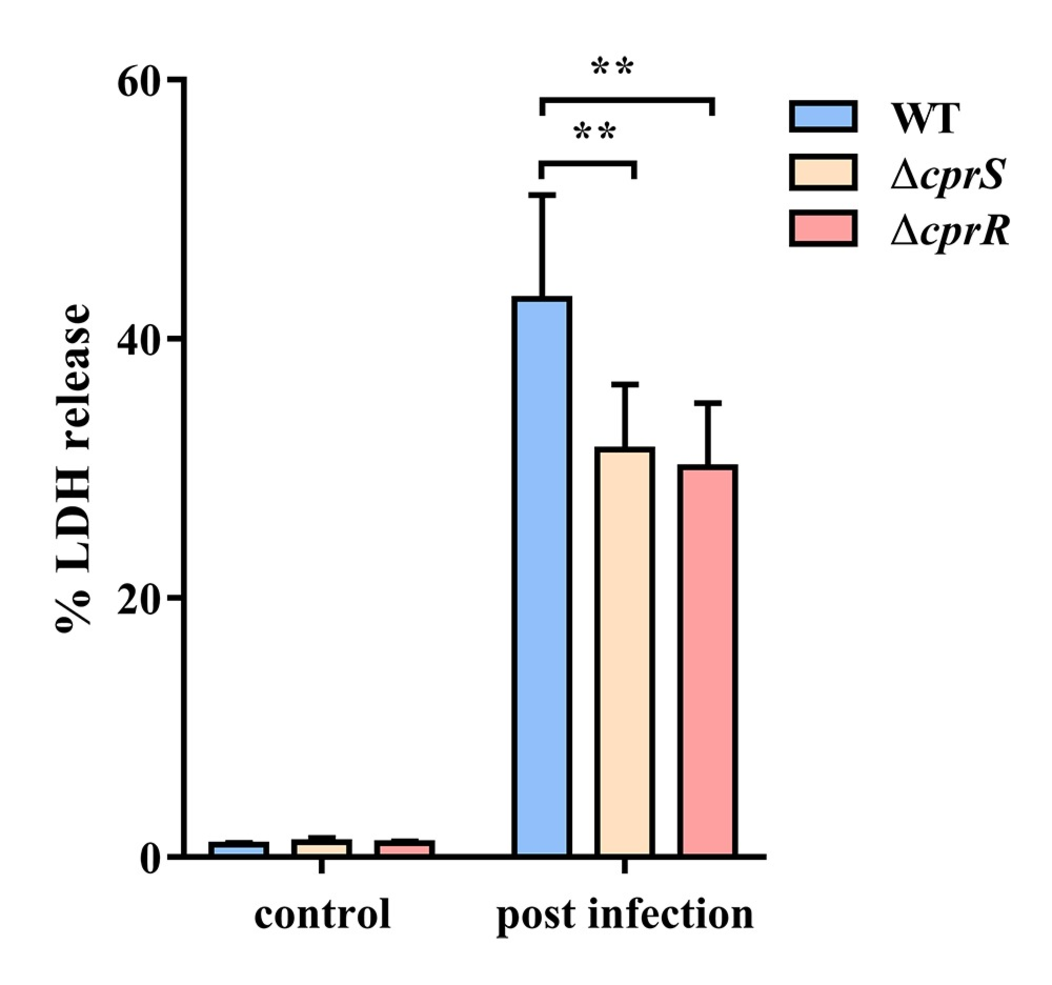

Supplement: S7 Fig — Raw264.7 cell were infected by different strains at an MOI of 10 for 4 h, the relative cytotoxicity was then determined by the LDH release assay. Error bars indicate the means ± SD of three independent experiments. *P <0.05; **P < 0.01; ***P < 0.001. (TIF) [file ppat.1011946.s007.tif]
