# Supplementary material for: Pseudomonas aeruginosa two-component system CprRS regulates HigBA expression and bacterial cytotoxicity in response to LL-37 stress
Source: PLoS Pathog. 2024 Jan 10;20(1):e1011946. doi: 10.1371/journal.ppat.1011946 (PMC10805311; doi:10.1371/journal.ppat.1011946)
Supplement: S1 Table — (DOCX) [file ppat.1011946.s008.docx]

**Table S1. List of genes with potential CprR-binding site in promoter regions.**

| **Predicted binding site** | **Locus in PAO1** | **Locus in PA14** | **Gene and function** |
| --- | --- | --- | --- |
| CTAACGTTAT | PA0226 | CIA_04857 | *bkdA1*, putative 3-oxoadipate-CoA transferase subunit A |
| TTAACGTTAA | PA4674 | CIA_00023 | *higB*, type II toxin-antitoxin system HigBA, virulence, biofilm formation, persistance |
