# Supplementary material for: Pseudomonas aeruginosa two-component system CprRS regulates HigBA expression and bacterial cytotoxicity in response to LL-37 stress
Source: PLoS Pathog. 2024 Jan 10;20(1):e1011946. doi: 10.1371/journal.ppat.1011946 (PMC10805311; doi:10.1371/journal.ppat.1011946)
Supplement: S2 Table — (DOCX) [file ppat.1011946.s009.docx]

**Table S2. Significant downregulated proteins in Δ*cprR*Δ*higB* compared with WT after LL-37 treatment.**

| **Locus in PAO1** | **Locus in PA14** | **Protein name and Functions** | **Fold changes (log_2_) Δ*cprR*Δ*higB* /WT** | **Student's T-test *p*-value** |
| --- | --- | --- | --- | --- |
| PA4636 | CIA_00063 | Phospholipid/glycerol acyltransferase domain-containing protein | -1.83578 | 0.014229 |
| PA4633 | CIA_00068 | methyl-accepting chemotaxis protein | -1.1171 | 0.006327 |
| PA4619 | CIA_00084 | c-type cytochrome CBB3 | -1.88204 | 0.018501 |
| PA4613 | CIA_00091 | Catalase KatB | -1.00006 | 0.000614 |
| PA4470 | CIA_00346 | Fumarate hydratase FumC2 | -1.98099 | 0.038121 |
| PA4466 | CIA_00350 | Phosphocarrier protein HPr | -3.14558 | 0.00044 |
| PA4314 | CIA_00505 | Formyltetrahydrofolate deformylase PurU1 | -2.22609 | 0.049583 |
| PA0760 | CIA_00646 | FAD assembly factor SdhE | -1.1888 | 0.007414 |
| PA0859 | CIA_00753 | DSBA-like thioredoxin domain-containing protein | -2.38019 | 0.047334 |
| PA1086 | CIA_00990 | flagellar hook-associated protein FlgK | -1.36004 | 0.005685 |
| PA1087 | CIA_00991 | flagellar hook-associated protein FlgL | -1.66415 | 0.046763 |
| PA1141 | CIA_01047 | LysR family transcriptional regulator | -1.29018 | 0.021233 |
| PA1280 | CIA_01203 | Histidine phosphatase family protein | -1.77177 | 0.036666 |
| PA1293 | CIA_01216 | Strictosidine synthase conserved region domain-containing protein | -1.13515 | 0.042732 |
| PA1513 | CIA_01430 | Urate oxidase N-terminal domain-containing protein | -2.51738 | 0.001884 |
| PA1602 | CIA_01521 | Probable oxidoreductase | -1.09239 | 0.000212 |
| PA1840 | CIA_01777 | DUF1883 domain-containing protein | -1.80884 | 0.02286 |
| PA2379 | CIA_02316 | (2Fe-2S)-binding protein | -1.06346 | 0.003981 |
| PA2463 | CIA_02408 | two-partner secretion system transporter TpsB1 | -1.66499 | 0.009902 |
| PA2482 | CIA_02427 | cytochrome C | -1.45882 | 0.019525 |
| PA2611 | CIA_02609 | sirohydrochlorin ferrochelatase | -1.64952 | 0.046375 |
| PA2627 | CIA_02625 | DNA repair protein HhH-GPD | -1.94022 | 0.001782 |
| PA2729 | CIA_02730 | hypothetical protein | -2.88051 | 4.62E-05 |
|  | CIA_02736 | DNA helicase | -1.14676 | 0.022018 |
| PA2813 | CIA_02833 | glutathione S-transferase | -1.07521 | 0.025594 |
| PA3111 | CIA_03149 | folylpolyglutamate synthetase FolC | -1.86127 | 0.024541 |
| PA3247 | CIA_03304 | M18 aminopeptidase in mucin utilization ApeB | -2.08025 | 0.007204 |
| PA3344 | CIA_03408 | ATP-dependent DNA helicase RecQ | -1.69691 | 0.025249 |
| PA3351 | CIA_03415 | anti-sigma-28 factor FlgM | -1.10283 | 0.002094 |
| PA3601 | CIA_03657 | 50S ribosomal protein L31 | -1.68471 | 0.026029 |
| PA3765 | CIA_03677 | ferredoxin | -1.14797 | 0.002053 |
| PA3757 | CIA_03813 | GntR family transcriptional regulator | -1.93388 | 0.002136 |
| PA3764 | CIA_03820 | lytic transglycosylase, MltF | -2.05694 | 0.017052 |
| PA3765 | CIA_03821 | hypothetical protein | -1.23959 | 0.036189 |
|  | CIA_03834 | lactate dehydrogenase | -2.60031 | 0.001833 |
| PA3787 | CIA_03877 | M23 family metallopeptidase | -1.19047 | 0.035798 |
| PA3917 | CIA_04014 | molybdopterin converting factor, small subunit MoaD | -1.30333 | 0.020821 |
| PA3967 | CIA_04067 | hypothetical protein | -1.58978 | 0.00688 |
| PA4055 | CIA_04155 | riboflavin synthase alpha chain RibC | -1.33143 | 0.000977 |
| PA4063 | CIA_04162 | hypothetical protein | -1.01689 | 0.029857 |
| PA4198 | CIA_04297 | acyl-CoA synthetase | -2.09203 | 0.037652 |
| PA4225 | CIA_04324 | pyochelin synthetase pchF | -1.04298 | 0.026594 |
| PA4229 | CIA_04328 | pyochelin biosynthetic protein PchC | -1.73261 | 0.007797 |
| PA4278 | CIA_04381 | sporulation protein | -1.046 | 0.019629 |
| PA0580 | CIA_04473 | O-sialoglycoprotein endopeptidase gcp | -1.0986 | 0.030698 |
|  | CIA_04753 | transcriptional regulator | -2.21591 | 0.032227 |
| PA5406 | CIA_05246 | hypothetical protein | -1.3703 | 0.000816 |
| PA5221 | CIA_05439 | FAD-dependent monooxygenase | -1.95716 | 0.012221 |
| PA5107 | CIA_05559 | Outer membrane lipoprotein Blc | -2.71625 | 0.000521 |
| PA5050 | CIA_05617 | primosome assembly protein PriA | -2.33751 | 0.031069 |
| PA4991 | CIA_05676 | FAD-dependent oxidoreductase | -1.49138 | 0.046472 |
| PA4708 | CIA_05972 | Heme-transport protein, PhuT | -1.14936 | 0.014213 |
