# Supplementary material for: Pseudomonas aeruginosa two-component system CprRS regulates HigBA expression and bacterial cytotoxicity in response to LL-37 stress
Source: PLoS Pathog. 2024 Jan 10;20(1):e1011946. doi: 10.1371/journal.ppat.1011946 (PMC10805311; doi:10.1371/journal.ppat.1011946)
Supplement: S3 Table — (DOCX) [file ppat.1011946.s010.docx]

**Table S3. Significant downregulated proteins in Δ*cprS*Δ*higB* compared with WT after LL-37 treatment.**

| **Locus in PAO1** | **Locus in PA14** | **Protein name and Functions** | **Fold changes (log_2_) Δ*cprS*Δ*higB* /WT** | **Student's T-test *p*-value** |
| --- | --- | --- | --- | --- |
| PA4587 | CIA_00084 | Cytochrome c551 peroxidase CcpA | -1.68564 | 0.018404 |
| PA4614 | CIA_00090 | large conductance mechanosensitive channel protein MscL | -1.49602 | 0.009254 |
| PA4587 | CIA_00118 | cytochrome C biogenesis protein CcsA | -1.43591 | 0.000956 |
| PA4476 | CIA_00340 | TIGR02099 family protein | -3.93684 | 0.001697 |
| PA4466 | CIA_00350 | Phosphocarrier protein PtsH | -2.19512 | 0.008682 |
| PA4446 | CIA_00371 | periplasmic serine endoprotease AlgW | -1.23348 | 0.002113 |
| PA4426 | CIA_00391 | secreted lipoprotein | -1.42168 | 2.44E-07 |
| PA4321 | CIA_00498 | DUF4350 domain-containing protein | -1.31779 | 1.72E-05 |
| PA0672 | CIA_00547 | Heme oxygenase PigA | -1.16557 | 2.52E-05 |
| PA0779 | CIA_00666 | Lon protease Lon | -3.56925 | 0.026982 |
| PA0793 | CIA_00682 | 2-methylaconitate cis-trans isomerase PrpF | -1.9705 | 0.002058 |
|  | CIA_00715 | hypothetical protein | -3.25451 | 1.03E-05 |
| PA0836 | CIA_00730 | acetate kinase | -1.06626 | 0.017918 |
| PA0926 | CIA_00825 | acetate kinase | -4.03466 | 0.000956 |
| PA0949 | CIA_00849 | Trp repressor binding protein WrbA | -2.49097 | 0.018029 |
|  | CIA_00887 | Type III secretion system effector ExoU | -1.47868 | 0.02607 |
| PA4755 | CIA_00929 | Transcription elongation factor GreA | -1.75534 | 0.023901 |
| PA1127 | CIA_01033 | general stress protein | -2.15164 | 1.74E-05 |
|  | CIA_01057 | pyoS3A | -1.17278 | 0.006995 |
| PA1315 | CIA_01148 | TetR family transcriptional regulator | -1.94937 | 0.029066 |
| PA1272 | CIA_01195 | cob(I)alamin adenolsyltransferase/cobinamide ATP-dependent adenolsyltransferase | -1.77031 | 8.80E-05 |
| PA1431 | CIA_01348 | regulatory protein RsaL | -1.17503 | 0.001921 |
| PA1457 | CIA_01374 | chemotaxis protein CheZ | -1.76799 | 4.77E-05 |
| PA1480 | CIA_01396 | heme lyase subunit CcmF | -2.0021 | 0.008175 |
| PA1513 | CIA_01430 | membrane protein | -1.51364 | 0.001586 |
| PA1528 | CIA_01445 | cell division protein ZipA | -1.13491 | 0.000843 |
| PA1552 | CIA_01473 | cytochrome CBB3 | -1.13707 | 0.003817 |
| PA1593 | CIA_01512 | thioesterase | -1.13793 | 0.003322 |
| PA1602 | CIA_01521 | (2Fe-2S)-binding protein | -1.47728 | 0.025016 |
| PA1608 | CIA_01527 | methyl-accepting chemotaxis protein | -2.66408 | 0.021293 |
| PA1723 | CIA_01650 | type III export protein PscK | -1.37547 | 1.05E-06 |
| PA1724 | CIA_01651 | type III export protein PscL | -1.58808 | 0.011295 |
| PA1768 | CIA_01695 | hypothetical protein | -1.16436 | 0.025171 |
| PA1795 | CIA_01722 | cysteinyl-tRNA synthetase | -1.0315 | 0.001803 |
| PA1832 | CIA_01767 | peptidase | -1.54981 | 0.000255 |
| PA1999 | CIA_01937 | succinyl-CoA:3-ketoacid-CoA transferase | -2.89049 | 0.007853 |
| PA2081 | CIA_02019 | kynurenine formamidase KynB | -2.58552 | 0.000324 |
| PA2082 | CIA_02020 | AsnC family transcriptional regulator KynR | -1.32387 | 0.000487 |
| PA2261 | CIA_02203 | 2-dehydro-3-deoxygluconokinase | -2.75858 | 1.34E-05 |
| PA2273 | CIA_02215 | Redox-sensitive transcriptional activator SoxR | -3.77824 | 2.36E-05 |
| PA2290 | CIA_02232 | glucose dehydrogenase | -1.11397 | 3.07E-06 |
| PA2379 | CIA_02316 | (2Fe-2S)-binding protein | -2.16206 | 0.001137 |
| PA2482 | CIA_02427 | cytochrome C | -1.34764 | 0.018419 |
|  | CIA_02733 | DEAD/DEAH box helicase | -3.1891 | 0.006871 |
|  | CIA_02736 | DNA helicase | -1.09719 | 0.002899 |
|  | CIA_02754 | hypothetical protein | -1.22596 | 7.66E-05 |
| PA2841 | CIA_02872 | enoyl-CoA hydratase | -3.57383 | 0.000775 |
| PA2859 | CIA_02890 | transcription elongation factor GreB | -2.0051 | 2.85E-05 |
| PA2932 | CIA_02964 | morphinone reductase morB | -2.03219 | 0.00321 |
| PA2954 | CIA_02989 | hypothetical protein | -1.4114 | 0.009584 |
| PA2986 | CIA_03021 | lipoprotein localization protein LolC | -1.08589 | 0.049375 |
| PA3055 | CIA_03091 | hypothetical protein | -1.22126 | 0.015745 |
| PA3179 | CIA_03214 | ribosomal large subunit pseudouridine synthase B | -2.11498 | 1.01E-05 |
| PA3221 | CIA_03257 | tRNA-binding protein | -1.06929 | 0.000655 |
| PA3299 | CIA_03361 | long-chain-fatty-acid--CoA ligase FadD1 | -1.9666 | 0.002068 |
| PA3351 | CIA_03415 | anti-sigma-28 factor FlgM | -1.35882 | 2.01E-06 |
| PA3468 | CIA_03540 | mechanosensitive ion channel protein MscS | -1.46371 | 9.89E-05 |
| PA3470 | CIA_03542 | NUDIX hydrolase | -1.26945 | 0.002416 |
| PA3563 | CIA_03617 | transcriptional regulator | -2.07737 | 0.002725 |
| PA3621 | CIA_03677 | ferredoxin | -1.80451 | 2.92E-05 |
| PA3626 | CIA_03682 | tRNA pseudouridine synthase D | -1.43154 | 0.005438 |
| PA3646 | CIA_03702 | UDP-3-O-(3-hydroxymyristoyl) glucosamine N-acyltransferase | -1.5653 | 0.017088 |
| PA3664 | CIA_03720 | ArsC family transcriptional regulator | -1.83446 | 0.033207 |
| PA3676 | CIA_03732 | Resistance-Nodulation-Cell Division (RND) efflux transporter MexK | -2.52084 | 1.93E-05 |
| PA3699 | CIA_03755 | TetR family transcriptional regulator | -3.43977 | 4.99E-05 |
| PA3716 | CIA_03772 | hypothetical protein | -1.10139 | 0.007163 |
| PA3765 | CIA_03821 | hypothetical protein | -1.85386 | 0.009403 |
| PA3787 | CIA_03877 | peptidase M23 | -2.09494 | 0.007603 |
| PA3789 | CIA_03879 | peptidase | -1.12366 | 0.000105 |
| PA3815 | CIA_03905 | transcriptional regulator | -1.16931 | 0.002889 |
| PA3871 | CIA_03965 | peptidyl-prolyl cis-trans isomerase | -1.07401 | 0.028061 |
| PA3878 | CIA_03972 | two-component sensor NarX | -1.05784 | 0.003261 |
| PA3915 | CIA_04012 | molybdopterin biosynthesis protein B | -1.25999 | 0.000876 |
| PA3917 | CIA_04014 | molybdenum cofactor biosynthesis protein MoaD | -1.65276 | 0.008815 |
| PA3421 | CIA_04022 | hypothetical protein | -1.49467 | 0.000251 |
| PA3978 | CIA_04080 | hypothetical protein | -4.57617 | 3.03E-07 |
| PA4055 | CIA_04155 | riboflavin synthase subunit alpha | -2.16173 | 0.007415 |
| PA4198 | CIA_04297 | acyl-CoA synthetase | -2.18998 | 0.025726 |
| PA4207 | CIA_04306 | acriflavine resistance protein B | -1.18976 | 0.002566 |
| PA0542 | CIA_04514 | hypothetical protein | -1.42466 | 0.001858 |
| PA0520 | CIA_04537 | regulatory protein NirQ | -1.46483 | 0.00093 |
| PA0516 | CIA_04541 | heme bioge | -1.5417 | 0.000792 |
| PA0494 | CIA_04561 | acetyl-CoA carboxylase biotin carboxylase subunit | -1.73886 | 0.034735 |
| PA0315 | CIA_04743 | rod shape-determining protein RodA | -1.42304 | 0.001218 |
|  | CIA_04753 | transcriptional regulator | -1.16697 | 0.049976 |
| PA0261 | CIA_04820 | Type VI secretion system protein Tli3 | -1.27334 | 0.0265 |
| PA0259 | CIA_04822 | Type VI secretion system protein Tla3 | -1.92033 | 0.041003 |
| PA0125 | CIA_04951 | antitoxin ParD | -1.19689 | 0.000288 |
| PA0122 | CIA_04954 | Hemolysin RahU | -3.48703 | 0.003048 |
| PA0079 | CIA_04998 | type VI secretion protein TssK1 | -2.41502 | 0.000864 |
| PA5565 | CIA_05085 | tRNA uridine 5-carboxymethylaminomethyl modification protein | -1.59228 | 0.036283 |
| PA5529 | CIA_05132 | sodium:proton antiporter | -2.25154 | 0.004911 |
| PA5492 | CIA_05158 | GTP-binding protein YsxC | -2.3693 | 0.004428 |
| PA5438 | CIA_05212 | RpiR family transcriptional regulator | -1.4417 | 0.000612 |
| PA5285 | CIA_05371 | hypothetical protein | -1.1602 | 8.01E-07 |
| PA5221 | CIA_05439 | 2-octaprenyl-3-methyl-6-methoxy-1,4-benzoquinol hydroxylase | -3.20191 | 0.000151 |
| PA5188 | CIA_05473 | 3-hydroxyacyl-CoA dehydrogenase | -2.49609 | 0.009928 |
| PA5135 | CIA_05531 | hypothetical protein | -1.06593 | 0.049938 |
| PA5077 | CIA_05590 | Glucans biosynthesis glucosyltransferase H OpgH | -1.2775 | 0.00783 |
| PA5045 | CIA_05622 | penicillin-binding protein 1A MrcA | -1.23595 | 0.035158 |
| PA5002 | CIA_05665 | de-N-acetylase involved in persistence, DnpA | -1.19405 | 0.003328 |
| PA4997 | CIA_05670 | transport protein MsbA | -3.58567 | 0.019867 |
| PA4957 | CIA_05711 | Phosphatidylserine decarboxylase proenzyme psd | -1.29358 | 0.00092 |
| PA4846 | CIA_05825 | 3-dehydroquinate dehydratase AroQ1 | -1.32668 | 0.012514 |
| PA4708 | CIA_05972 | Heme-transport protein, PhuT | -1.37067 | 0.001275 |
