# Supplementary material for: Pseudomonas aeruginosa two-component system CprRS regulates HigBA expression and bacterial cytotoxicity in response to LL-37 stress
Source: PLoS Pathog. 2024 Jan 10;20(1):e1011946. doi: 10.1371/journal.ppat.1011946 (PMC10805311; doi:10.1371/journal.ppat.1011946)
Supplement: S4 Table — (DOCX) [file ppat.1011946.s011.docx]

**Table S4. Bacteria strains and plasmids.**

| **Reagent or Resource** | **Source** | **Identifier** |
| --- | --- | --- |
| **Bacterial cells and plasmids** |  |  |
| *E. coli* BL21(DE3) | Beijing Genesand Biotech Co.,Ltd | Cat# SEC19 |
| *E. coli* DH5α | Beijing Genesand Biotech Co.,Ltd | Cat# SCC01 |
| *P. aeruginosa* PA14 | Prof. Wang’ Lab | N/A |
| *P. aeruginosa* Δ*cprS* | This study |  |
| *P. aeruginosa* Δ*cprR* | This study |  |
| *P. aeruginosa* Δ*cprS*Δ*higB* | This study |  |
| *P. aeruginosa* Δ*cprR*Δ*higB* | This study |  |
| pME6032 | Previous study | Song et al., 2021 |
| pEX18-Gm | Previous study | Song et al., 2021 |
| pRG970-Km | Previous study | Song et al., 2021 |
