# Supplementary material for: Pseudomonas aeruginosa two-component system CprRS regulates HigBA expression and bacterial cytotoxicity in response to LL-37 stress
Source: PLoS Pathog. 2024 Jan 10;20(1):e1011946. doi: 10.1371/journal.ppat.1011946 (PMC10805311; doi:10.1371/journal.ppat.1011946)
Supplement: S5 Table — (DOCX) [file ppat.1011946.s012.docx]

**Table S5. Primers used in the work.**

| **Purpose/Name** | **Sequence (5'-3')** |
| --- | --- |
| **Protein Expression** | |
| **pET22b-*cprS*-f** | CTTTAAGAAGGAGATATACATATGATGAAACGCGGCCTGAGCC |
| **pET22b-*cprS*-r** | TGGTGGTGGTGCTCGAGGTCCGGCGTGGCGGGGAACAGCAGCT |
| **pET22b-*cprR*-f** | CTTTAAGAAGGAGATATACATATGATGCATATCCACGTACTCG |
| **pET22b-*cprR*-r** | GTGGTGGTGGTGCTCGAGTTTGCGCTCCAGCTGGAAGCCGACG |
| **pET22b-*cprS*_28-163_-f** | CACCACCACCACCACCCGCTGTCCGACGCCATCATCG |
| **pET22b- *cprS*_28-163_-r** | GTGGTGGTGGTGGTGGCCGTAGTCGCCGAGCATGCG |
| **pET22b-*cprR*_D53A-_f** | ATCATGCTGCCGCGCATCAAC |
| **pET22b-*cprR*_D53A_-r** | CGCCAGCAGGATCACGTCG |
| **pET22b-*cprR*_W180A-_f** | GGCGACGAACCGCCGGAAAGC |
| **pET22b-*cprR*_W180A_-r** | CGCGACGCTGCGCCCCAGCTGC |
| **pET22b-*cprR*_S187A-_f** | AACACCCTCAACGTGCACATG |
| **pET22b-*cprR*_S187A_-r** | CGCTTCCGGCGGTTCGTCGCC |
| **pET22b-*cprR*_N188A-_f** | ACCCTCAACGTGCACATGC |
| **pET22b-*cprR*_N188A_-r** | CGCGCTTTCCGGCGGTTCGTCG |
| **pET22b-*cprR*_V192A-_f** | CACATGCACCACCTGCGCAG |
| **pET22b-*cprR*_V192A_-r** | CGCGTTGAGGGTGTTGCTTTCC |
| **pET22b-*cprR*_R198A-_f** | AGCACCGTCGACAAGG |
| **pET22b-*cprR*_R198A_-r** | CGCCAGGTGGTGCATGTGCACG |
| **pET22b-*cprR*_H196A-_f** | TAATTAACCTAGGCTGCTGC |
| **pET22b-*cprR*_H196A_-r** | CGCGTGCATGTGCACGTTGAG |
| **pET22b-*cprR*_H214A-_f** | AGCGTCGGCTTCCAGCTG |
| **pET22b-*cprR*_H214A_-r** | CGCCAGGGTATGGATCAGAGG |
| **Gene knockout** | |
| **pEX18-*cprS*-upstream-f** | AACGACGGCCAGTGCCAAGCTTGGCCGCCGGGGTGGTCTGCG |
| **pEX18-*cprS*-upstream-r** | CTCCTTCTTAAAGTTAAACTTGCGCTCCAGCTGGAAGC |
| **pEX18-*cprS*-downstream-f** | GTTTAACTTTAAGAAGGAGAGCGCGACGGCCTTCCTC |
| **pEX18-*cprS*-downstream-r** | TTCGAGCTCGGTACCCGGGGATACGAGGTGATCAAGCAGTCC |
| **pEX18-*cprR*-upstream-f** | AACGACGGCCAGTGCCAAGCTTTGGTCGAGCCCGGCAAGAG |
| **pEX18-*cprR*-upstream-r** | CTCCTTCTTAAAGTTAAACGGTATCTGTTCGCATGACG |
| **pEX18-*cprR*-downstream-f** | GTTTAACTTTAAGAAGGAGAACGCGGCCTGAGCCTGATC |
| **pEX18- *cprR* -downstream-r** | TTCGAGCTCGGTACCCGGGGATCGAGGTCGTCGAGGGTCATC |
| **pEX18-*higB*-upstream-f** | AACGACGGCCAGTGCCAAGCTTGTACGACCCTGGGAGCCGATATCC |
| **pEX18-*higB*-upstream-r** | CTCCTTCTTAAAGTTAAACTCATTAACCCTTAACGTTAAG |
| **pEX18-*higB*-downstream-f** | GTTTAACTTTAAGAAGGAGATGGCTACCAATGGTATGC |
| **pEX18-*higB*-downstream-r** | TTCGAGCTCGGTACCCGGGGATGATCAGAAAATTATCTAGC |
| **qRT-PCR** | |
| **18s RNA-f** | CTCAACACGGGAAACCTCAC |
| **18s RNA-r** | CGCTCCACCAACTAAGAACG |
| **IL-1β-f** | GGGACTTGAAGAGAGAAGTGG |
| **IL-1β-r** | CTTTCCCTTGATCCCTAAGGT |
| **IL-8-f** | GATTGCTCAACCTTTTCGCATTAC |
| **IL-8-r** | CATGGCCTGTGATTTAGCTGTG |
| **IL-12-f** | GAAGACCGTAAGGACCTGTG |
| **IL-12-r** | TTGCAAAGTAGTAGTGCGGAG |
| **TNF-α-f** | CCCCCAGAAGGAAGAGTTTC |
| **TNF-α*-*r** | CGGGCTTATCTGAGGTTTGA |
| **Reporter Plasmid** | |
| **pRG970-P*_higB_*-f** | GACTGACCTACCCGGGGATCCTAACCCTTAACGTTAAGCGT |
| **pRG970-P*_higB_*-r** | CTCTAGAAGAAGCTTGGGATCCGAGGAGATTTCCGAGTTGC |
| **pRG970-seqencing-F** | ATTCAGGCTGCGCAACTG |
| **EMSA** |  |
| **P*_higB_*-f** | CTGGTCGAGTCGATGATGG |
| **P*_higB_*-r** | GGCAACCTCACCAGACGATA |
| **P*_fadD6_*-f** | ACGCCGGTCAGGCTGCCCTG |
| **P*_fadD6_*-r** | ACACCTGGGAGCGGGTTCGT |
